# Gene regulatory network analysis of silver birch reveals the ancestral state of secondary cell wall biosynthesis in core eudicots

**DOI:** 10.1111/nph.70126

**Published:** 2025-04-16

**Authors:** Maja Ilievska, Sun‐Li Chong, Kean‐Jin Lim, Juha Immanen, Kaisa Nieminen, Hannu Maaheimo, Yrjö Helariutta, Joel Wurman‐Rodrich, Paul Dupree, James Ord, Maija Tenkanen, Jarkko Salojärvi

**Affiliations:** ^1^ Faculty of Biological and Environmental Sciences, Organismal and Evolutionary Biology Research Programme and Viikki Plant Science Centre University of Helsinki Helsinki FI‐00014 Finland; ^2^ Department of Food and Nutrition University of Helsinki Helsinki FI‐00014 Finland; ^3^ State Key Laboratory of Subtropical Silviculture, College of Forestry and Biotechnology Zhejiang A&F University Hangzhou 311300 China; ^4^ Natural Resources Institute Finland (Luke) Helsinki FI‐00791 Finland; ^5^ VTT Technical Research Centre PO Box 1000 Espoo FI‐02044 Finland; ^6^ Sainsbury Laboratory University of Cambridge Cambridge CB2 1LR UK; ^7^ Department of Biochemistry University of Cambridge Cambridge CB2 1QW UK; ^8^ School of Biological Sciences Nanyang Technological University Singapore 637551 Singapore; ^9^ Singapore Centre for Environmental Life Sciences Engineering Nanyang Technological University Singapore 637551 Singapore

**Keywords:** *Betula pendula* (silver birch), gene regulation, genome evolution, transcriptomics, wood development

## Abstract

- The compact genome and lack of recent whole‐genome multiplication (WGM) events make the boreal pioneer tree silver birch (*Betula pendula*) a promising model for primary and secondary cell wall (PCW and SCW) regulation in forest trees.
- Here, we constructed regulatory networks through combined co‐expression and promoter motif analysis and carried out a tissue‐wide analysis of xylan using mass spectrometry.
- Analyses confirm the evolutionarily conserved model of superimposed layers of regulation and suggest a relatively simple ancestral state still retained in birch. Multispecies network analysis, including birch, poplar, and eucalyptus, identified conserved regulatory interactions, highlighting lignin biosynthesis as least conserved. The SCW biosynthesis co‐expression module was enriched with WGM duplicates. While regulator genes were under positive selection, others evolved under relaxed purifying selection, possibly linked with diversification, as indicated by expression and regulatory motif differences. Xylan composition varied between PCW and SCW, revealing unique acetylation patterns. PCW xylan biosynthesis genes showed distinct expression and regulatory motifs, with a novel acetyl transferase potentially involved.
- This work highlights birch as a valuable model for understanding wood formation, vascular development, and cell wall composition in eudicots.

## Introduction

The plant cell wall (CW) was a key evolutionary innovation for vascular plants, enabling their colonization of terrestrial ecosystems. During secondary growth, the CW forms from the vascular cambium, beginning with primary cell wall (PCW) synthesis in young xylem and phloem cells, followed by secondary cell wall (SCW) deposition in specialized cells like tracheary elements and fibers. The SCW provides structural support, protection from pathogens, and aids in water and nutrient transport. Secondary cell wall consists of cellulose microfibrils in a matrix of lignin and hemicelluloses, mainly xylan. Understanding xylan structure and regulation is crucial for advancing lignocellulosic biotechnology (Chandel *et al*., 2011; Xiong *et al*., 2013).

**Fig. 1:**
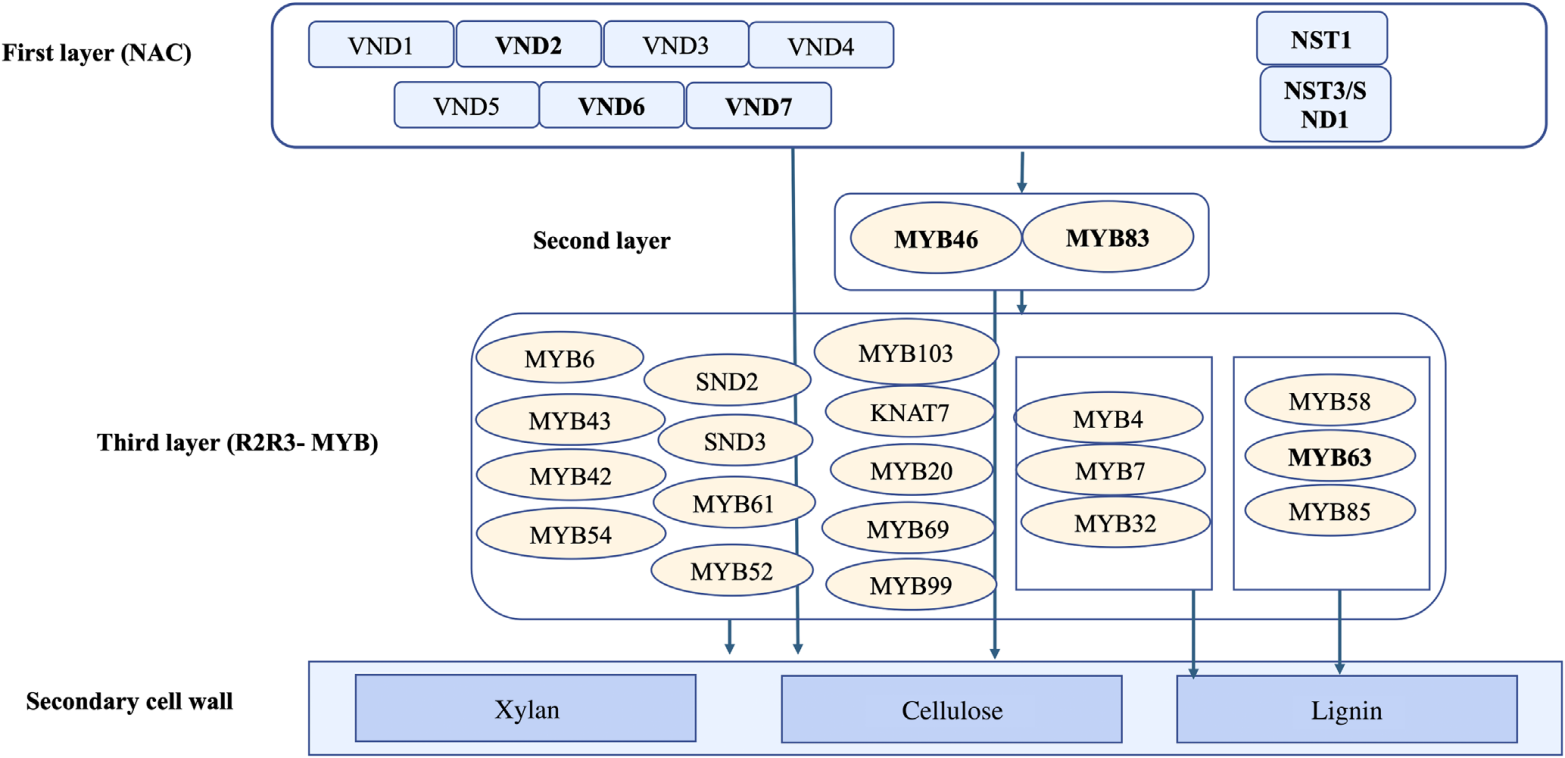
Three layers of regulation of cell wall biosynthesis in *Arabidopsis thaliana* (Zhong *et al*., 2010; Nakano *et al*., 2015; Zhang *et al*., 2018) consist of NAC and MYB family transcription factors.

The SCW regulatory network, well studied in *Arabidopsis thaliana* (*Arabidopsis*), is highly conserved across angiosperms but remains understudied in woody species (Zhang *et al*., 2018; Li *et al*., 2024). In *Arabidopsis*, this network comprises three regulatory layers: first‐layer NAC master regulators (e.g. VND1‐7, NST1‐2, and SND1), as well as second‐layer (AtMYB46 and AtMYB83) and third‐layer Myeloblastosis (MYB) transcription factors (TFs; Mitsuda *et al*., 2005; Ko *et al*., 2014; Nakano *et al*., 2015; Zhang *et al*., 2018). These regulators control lignin, xylan, and cellulose biosynthesis, with overlapping and species‐specific roles (Fig. 1). Xylan biosynthesis involves GT47 xylosyltransferases and is partially regulated by second‐layer TFs, though regulators of xylan modifications remain unknown. Variations in the SCW regulatory networks among woody plants are particularly interesting due to their contribution to terrestrial biomass and their ecological value.

Gene dosage sensitivity indicates that certain genes are highly sensitive to changes in copy number, which can result in impaired fitness (Birchler & Veitia, 2007). Genes involved in signal transduction or transcription are especially dosage‐sensitive and thus are often retained after whole‐genome multiplication (WGM), driving evolution by introducing new traits and increasing complexity (Van de Peer *et al*., 2009; Ohno, 2013). Whole‐genome multiplication with dosage sensitivity and functional divergence delays reversion to singleton status, though gradual reversion is eventually favored (Conant *et al*., 2014). Syntenic gene pairs, or syntelogs, derived from a common ancestral region, provide insights into post‐WGM functional divergence, such as subfunctionalization, neofunctionalization, or hypofunctionalization – where reduced expression requires both syntelogs to maintain function (Lynch & Conery, 2000, Ohno, 2013, Birchler & Yang, 2022). Here, we explored the contribution of the gamma WGM event 125 million years ago (Ma), at the base of eudicots (Jiao *et al*., 2012), to the SCW gene diversification.

Silver birch (*Betula pendula*), a pioneer tree in boreal forests, is ideal for genetic studies due to its small genome (440 Mb) and lack of recent WGMs since the gamma event (Salojärvi *et al*., 2017). Since genes in a regulatory role are preferentially retained after WGMs (Carretero‐Paulet & Fares, 2012; Salojärvi *et al*., 2017), we hypothesized that the regulatory network of wood formation would involve fewer genes in birch compared with the current models *Arabidopsis* (with two Brassicaceae WGM events), *Populus trichocarpa* (one pan‐Salicaceae WGM event), or *Eucalyptus grandis (Eucalyptus)* (one WGM event shared across Myrtales), making it an attractive model for gene regulatory networks of wood formation in trees. Here, we aimed to map birch CW biosynthesis networks, compare them with *Arabidopsis*, *P. trichocarpa*, and *E. grandis*, and explore xylan biosynthesis and regulation using RNA‐sequencing data and chemical analysis of birch wood stem fractions.

## Materials and Methods

### Tissue collection and carbohydrate analysis

Mature stems of three clones of *Betula pendula* Roth (V5834) from the Viikki campus, University of Helsinki, were sampled. Phellem (tissue F1) was collected by peeling, followed by tangential cryosectioning for cork cambium (F2), old phloem (F3), young phloem (F4), vascular cambium (F5), young xylem (F6), old xylem (F7), and last year xylem (F8) using a cryotome (thickness: 10 μm, −27°C). The samples were cut into small pieces, freeze‐dried, and ground to fine powder using a bead mill. The alcohol‐insoluble residues (AIR) were prepared as described in Chong *et al*. (2014). Noncellulosic sugar compositions were quantified by acid methanolysis and gas chromatography (Chong *et al*., 2014).

### Phylogenetic inference

Proteomes were clustered using orthofinder (Emms & Kelly, 2019) with default settings. In addition to silver birch, we included *Amborella trichopoda* Baill (*Amborella*), monocot *Oryza sativa* L., and eudicots *Arabidopsis thaliana* Heynh., *Carica papaya* L., *Casuarina equisetifolia* L., *Eucalyptus grandis* W. Hill ex Maiden, *Fragaria vesca* L., *Medicago truncatula* Gaertn., *Populus trichocarpa* Torr. & A. Gray ex. Hook., *Quercus suber* L., *Solanum lycopersicum* L., *Theobroma cacao* L., *Trema orientalis* (L.) Blume, and *Vitis vinifera* L. *Gnetum montanum* Markgr. and *Picea abies* (L.) H. Karst. represented gymnosperms, and *Selaginella moellendorffii* Hieron. represented the lycophytes. Proteomes were downloaded from Plaza (Van Bel *et al*., 2018), and the remaining ones were from their project‐specific websites.

### Co‐expression clustering and multispecies network analysis

RNA was extracted from cryosectioned tissues (the fractions F1–F8 described above) and sequenced using an Illumina platform (Alonso‐Serra *et al*., 2019). The reads were aligned against the *B. pendula* transcriptome (Salojärvi *et al*., 2017) using kallisto v.0.43.0 (Bray *et al*., 2016); means of 4000 bootstrap replicates were obtained and transcripts per million (TPM)‐normalized (Alonso‐Serra *et al*., 2019). Poplar gene expression data were from Sundell *et al*. (2017), with 105–135 longitudinal cryo‐microtome sections across four *P. tremula* clonal replicates representing four developmental zones: phloem, cambium, early/developing xylem, and mature xylem. *Eucalyptus* gene expression data were obtained from Vining *et al*. (2015). The tissues included developing phloem, immature xylem, xylem, mature leaf, shoot tips, roots, young leaf, and flowers at different stages.

CLUST (Abu‐Jamous & Kelly, 2018) was performed with default settings and tested different tightness parameters. A signed similarity matrix was used for Weighted Gene Co‐expression Network Analysis (Langfelder & Horvath, 2008, Supporting Information Notes S1). The recommended pipeline yielded a soft thresholding power β = 10 and hierarchical clustering used Topological Overlap Measure. Eigengenes were used to calculate the correlation of the module with the other measurements. The cluster profiles were plotted in R.

### Gene Ontology enrichment

Gene Ontology (GO) enrichments were tested with goatools (Klopfenstein *et al*., 2018), using Bonferroni correction for multiple testing and adjusted *P*‐value < 0.05 as the threshold for significance. Gene Ontology annotations were obtained from the best BLAST hits to *Arabidopsis* using an E‐value threshold of 10^−4^. The GO terms were summarized with revigo (Supek *et al*., 2011). The GO terms selected for visualization had a threshold of −log_10_ (*P*‐val) > 2.1 corresponding to a *P*‐value < 0.008; only biological process terms were considered.

### Synteny analysis

coge synmap (Lyons *et al*., 2008) was used for synteny analyses. The number of synonymous mutations (*K*_s_) between syntelogs was used to estimate the relative age of divergence. Peaks in the histogram of *K*_s_ values between all syntelogs were used to identify the timing of WGM events. The ages of the duplication events were calibrated to the *K*_s_ peak of the gamma event.

Conservation of the binding sites was analyzed by testing the probability of observing the number of conserved motifs 1 K upstream of the transcription start site (TSS), between the syntelogs in the same cluster vs. syntelogs in different clusters and vs. those unclustered. Given that discrete data were used, with samples of different sizes and unequal variance, a one‐sided Wilcoxon test was used to test statistical significance.

### Transcription factor motif enrichment

Transcription factor binding sites in terms of position‐specific weight matrices were collected from Plant Cistrome (O'Malley *et al*., 2016) and JASPAR (Fornes *et al*., 2020). The SCW‐specific binding motifs, secondary wall NAC transcription factor‐binding element, and MYB regulatory motif (Zhong *et al*., 2010; Kim *et al*., 2012) were included in the analysis. FIMO tool from the MEME Suite was used to identify TF binding motifs 1000‐bp upstream of the TSSs. Each cluster was tested for motif enrichment using Fisher's exact test, and a false discovery rate adjusted *P*‐value < 0.05 was used as the threshold for significance. R scripts for enrichment analysis are available at Github, https://github.com/jsalojar/TFbindR.

### Cell wall biosynthesis regulatory network and inference of regulatory interactions

The regulatory network was constructed based on gene clusters enriched for SCW‐related GO processes, and the TFs whose binding motifs were enriched in those clusters. A regulatory interaction between the TF and the putative target gene was assumed if, in addition to the presence of the TF binding motif upstream of the target gene, the absolute Pearson correlation between the TF and the target was above 0.7 (using TPM values). Since the TFs were sometimes in a different cluster than the target, the screening was extended across all clusters using the same criteria. In the case of multiple potential regulators from the same family with the same binding motif, the regulator was chosen based on the highest correlation with the target.

Since the binding motifs of *KNAT7* and *KNAT3* are not known, target genes were identified with linear regression with the expression of target gene as the dependent variable and one or both TFs as covariates. The proportion of variance explained *R* ^2^ > 0.7 was used as the significance threshold for model selection. If at least one of the models was significant, the significantly best‐performing model was determined using the likelihood ratio test. Finally, GO enrichment analysis was carried out for the set of genes selected according to this procedure. The ‘arcdiagram’ package in R was used for network visualization.

To assess the conservation of network interactions, the same criteria of absolute correlation above 0.7 and the presence of the binding motif upstream 1 K of TSS were applied to both poplar and *Eucalyptus* expression networks.

### Isolation of xylans

The AIR samples were first treated sequentially by 50 mM 1,2‐diaminocyclohexanetetraacetic acid (CDTA), xyloglucanase of *Paenibacillus sp*. (Megazyme; dosage: 100 U g^−1^ AIR), and 11% peracetic acid for the removal of pectin, xyloglucan, and lignin, respectively. The holocellulose was incubated in dimethyl sulfoxide (DMSO) and shaken at 60°C overnight (Chong *et al*., 2014); the solid was centrifuged, and the solubilized acetylated xylans were precipitated in 80% EtOH and freeze‐dried. The solid remaining after DMSO treatment was incubated further in 4 M KOH and shaken at room temperature overnight to isolate deacetylated xylans. The supernatant was recovered by centrifugation, neutralized by formic acid, dialyzed against deionized water, and freeze‐dried.

### Xylans oligosaccharide mass profiling using AP‐MALDI‐ITMS and carbohydrate gel electrophoresis

Altogether, 1.5–3 mg of AIR were incubated in 20 mM sodium acetate, pH 5.0 containing GH10 endoxylanase of *Aspergillus aculeatus* (1200 U/g AIR; purified from Shearzyme, Novozyme) at 40°C for 24 h. The hydrolyzate was purified using a Graphitized carbon column (Chong *et al*., 2014). Before purification, 0.4 μg oxidized verbascose (α‐GalA‐(1→6)‐α‐Gal‐(1→6)‐α‐Gal‐(1→6)‐α‐Glc(1↔2)‐β‐Fru; gift from Dr Kirsti Parikka) was added as an internal standard. The oligosaccharide profiles were analyzed by atmospheric pressure‐matrix assisted laser desorption/ionization (AP‐MALDI)‐ion trap mass spectrometry (ITMS) (Chong *et al*., 2014). The mass list was exported and the intensity ratio for the selected peak to that of the internal standard was calculated and summed up as 100%. Subsequently, percentage of individual peak ratios was further analyzed using principal component analysis with SIMCA‐*P*+ 12.0.1 (Umetric, Umeå, Sweden). In addition, the AIR samples from young phloem and xylem were deacetylated with alkaline solution, digested with *Bo*GH30 xylanase, and the hydrolysates were then separated with polysaccharide analysis using carbohydrate gel electrophoresis (PACE) as performed previously in Wilson *et al*. (2022).

### Nuclear magnetic resonance spectroscopy analysis

The xylans were incubated in 50 mM sodium phosphate, pH 6, containing XynII endoxylanase of *Trichoderma reesei* (dosage: 1800 U g^−1^ xylans) at 40°C for 48 h. The insoluble solid was spun down, and the supernatant was collected, freeze‐dried, and exchanged one time in D_2_O. The quantitative heteronuclear single quantum coherence nuclear magnetic resonance (NMR) spectroscopy analysis was performed according to Chong *et al*. (2014).

## Results

### Gene co‐expression modules are linked with distinct biological processes

**Fig. 2:**
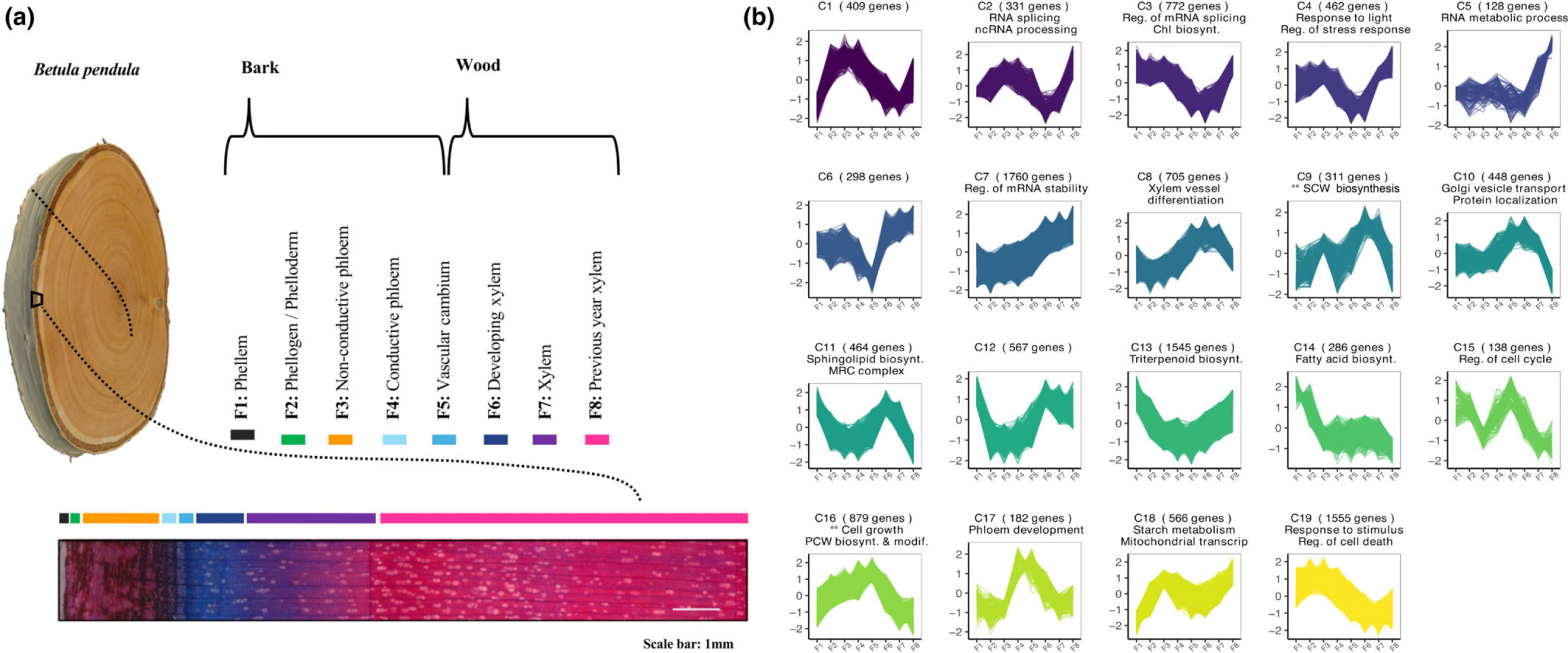
Cryosectioning of birch stem and the gene expression profiles of the identified clusters. (a) Cryosectioning of the birch stem into eight anatomically distinct tissues. Image adapted from Alonso‐Serra *et al*. (2019). (b) Gene expression profiles for each cluster. Expression values represent the average of replicates per fraction, with transcripts per million normalized using CLUST via quantile normalization, *z*‐score transformation, and log_2_ transformation. Cluster sizes are indicated in brackets, and the top enriched Gene Ontology (GO) biological processes are included. Biotsynth., biosynthesis; Modif., Modification; Reg., regulation; Transcript., transcription; Chl, chlorophyll. The ** mark the cell wall clusters.

**Fig. 3:**
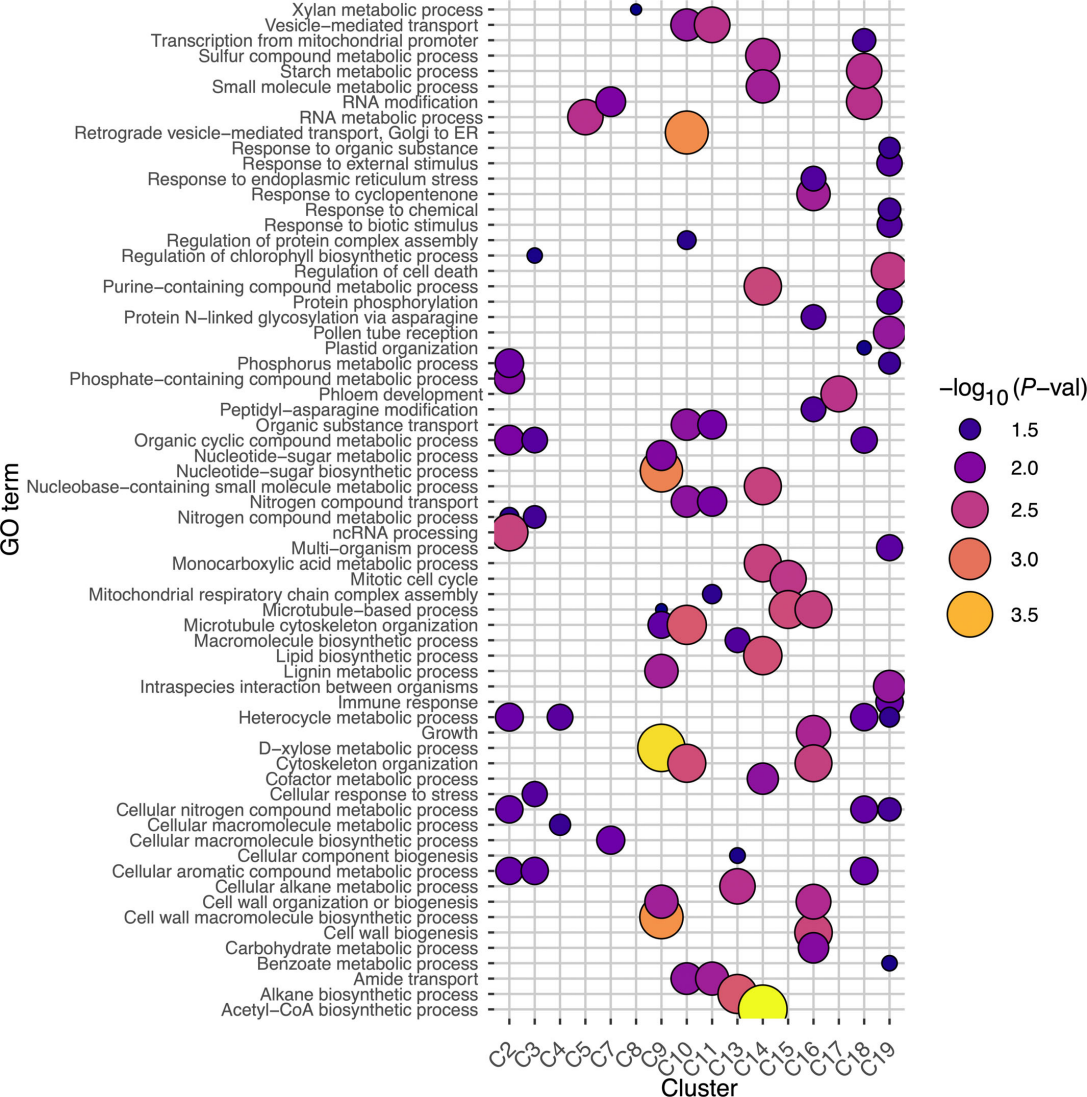
Gene Ontology (GO) enrichment analysis of the CLUST clusters for biological processes (BP). The *P*‐values were adjusted using Bonferroni correction and the significant GO terms (using a threshold of *P* < 0.008) were summarized with REVIGO (Supek *et al*., 2011). Clusters C1, C6 and C12 did not have any significant enrichment.

RNA sequencing and chemical composition were quantified from eight stem tissues (F1–F8, see Tissue collection, Alonso‐Serra *et al*., 2019; Fig. 2a). From the RNA‐sequencing data, genes were clustered into co‐expression modules using CLUST, which reduces cluster dispersion by limiting the number of genes in each cluster, ensuring high similarity in expression patterns (Abu‐Jamous & Kelly, 2018). The method grouped 11 806 genes into 19 tight clusters (128 to 1760 genes each), revealing tissue‐specific profiles and cluster‐specific enriched GO terms (Fig. 3; Notes S1; Tables S1–S3). Seven bark‐related clusters (1–4, 13, 14, and 19) peaked in F1–F4, with Clusters 3 and 4 enriched for genes involved in Chl biosynthesis and light response, likely reflecting photosynthesis in the birch phelloderm (Vandegehuchte *et al*., 2015; Alonso‐Serra *et al*., 2019). Cluster 14 was enriched for fatty acid biosynthesis, while Cluster 13, enriched for triterpenoid biosynthesis, contained orthologs involved in betulin and suberin production – compounds abundant in birch bark (Alonso‐Serra *et al*., 2019).

Cluster 9 was enriched for SCW biosynthesis (Tables S1, S4) and showed high activity in old phloem (F3) and developing xylem (F6) where SCW is deposited. Genes with high cluster membership were involved in generating CW polymer precursors, such as UDP‐xylose, regulation of cellulose integrity, secretion, and transport of CesA genes to the plasma membrane (Table S5). The cluster included orthologs of SCW regulators *AtNST1/NST2* and *AtSND1*.

Cluster 16, enriched for cell growth and CW modification, contained PCW genes involved in cellulose localization, cell growth (e.g. *EXPANSIN A1*), elongation, and pectin lyases. Cluster 8, peaking in developing xylem, was enriched for vessel differentiation and xylan biosynthesis, including SCW xylan‐associated genes and glycosyl hydrolases for xylan degradation (Fig. 2b; Table S5). Clusters 10 and 11, with expression peaks in cambium and developing xylem, were linked to CW biosynthesis and enriched for protein localization and endoplasmic reticulum (ER)‐to‐Golgi transport, essential for hemicelluloses and pectin biosynthesis (Alberts *et al*., 2002).

### SCW biosynthesis genes are augmented by WGM‐derived duplicates, with gamma duplicates involved in xylan, lignin, and CW modifications

Genes in syntenic genomic blocks demonstrated correlated expression patterns (Davidson *et al*., 2012), and we found them enriched among tissue‐specific co‐expressed genes (Alonso‐Serra *et al*., 2019). Here, we aimed to identify WGM and tandem duplicates across the co‐expression clusters, focusing on CW biosynthesis genes, especially the retention of gamma syntelogs, the expression divergence of the duplicates, and their contribution to functional diversification.

**Fig. 4:**
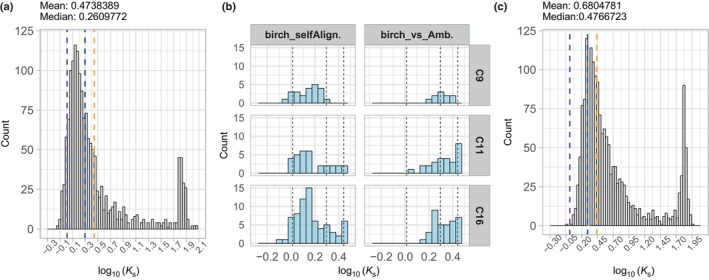
Analysis of syntenic gene pairs. (a) Histogram of the distribution of synonymous mutation rates (*K*_s_, log_10_‐transformed) in syntenic gene pairs based on birch self‐alignment. Lower *K*_s_ values on the left indicate younger syntelogs, while higher values represent older gene pairs. The estimated log_10_ (*K*_s_) peak for the core eudicot‐wide gamma duplication is *c*. 0.01283722 (Jiao *et al*., 2012), marked with a purple dashed line. Ancient genome duplications inferred from *Amborella trichopoda* paralogs suggest angiosperm‐wide and seed plant‐wide whole‐genome duplication (WGD) peaks at log_10_ (*K*_s_) = 0.2955891 and 0.4415852, respectively,marked with blue and orange dashed lines. The peak at *c*. log_10_(*K*_s_) of 1.8 represents misaligned duplicates. (b) Log_10_ (*K*_s_) distribution for Clusters 9, 11, and 16, which are enriched for WGD events retained in birch. The corresponding WGD *K*_s_ peaks are marked with vertical dashed lines. Log_10_ (*K*_s_) values are thresholded at 0.5. (c) Histogram of log_10_‐transformed *K*_s_ values for syntenic gene pairs based on the birch‐*Amborella* alignment. The gamma, angiosperm‐wide and seed plant‐wide WGDs are represented the purple, blue and orange dashed lines, respectively, reflecting common duplication events shared between these species.

Based on the synonymous substitution spectra (*K*_s_ values), birch syntelogs retained from the gamma triplication were more abundant than the duplicates from the more ancient WGMs (Fig. 4a). Clusters 9, 11, and 16 were enriched for syntelogs associated with processes related to CW biosynthesis, anthocyanin regulation, and cell cycle (Fisher exact test, *P*‐value < 0.05; Fig. 4b). By contrast, Clusters 7, 13, 17, and 19 were enriched for tandem duplicates, involved in stress responses and protein phosphorylation (Tables S6, S7). This supports the dosage balance hypothesis (Freeling & Thomas, 2006), which suggests the preferential retention of polyploid duplicates to be linked with regulation, while tandem duplicates contribute to recent environmental adaptations (Carretero‐Paulet & Fares, 2012; Salojärvi *et al*., 2017).

To contextualize the birch syntelogs within the broader framework of angiosperms, we conducted synteny analysis that included *A. trichopoda*, representing a basal angiosperm state before the gamma WGM, alongside *E. grandis* (postgamma duplication 109.9 Ma, Myburg *et al*., 2014), *Vitis vinifera* (no postgamma WGMs), *P. trichocarpa* (one whole‐genome duplication (WGD) 58 Ma), and *Arabidopsis* (two recent WGMs). Syntenic alignments against the *Amborella* genome revealed that over 80% of duplicates had reverted to a single copy in birch, *Vitis*, and *Eucalyptus*. Although *Arabidopsis* displayed a similar trend, *poplar* retained 39% of its syntelogs with a 2 : 1 relationship to *Amborella*, presumably reflecting the later Salicoid WGM in this lineage (Fig. S1). When comparing birch and *Eucalyptus*, out of 9128 syntelogs, 6991 had a 1 : 1 relationship, while 1324 birch genes had two copies in *Eucalyptus*, compared with 486 *Eucalyptus* genes having two copies in birch. Therefore, while *Eucalyptus* has largely reverted to a diploid state since its most recent WGD, it still retains a somewhat greater level of redundancy than in birch.

*Amborella* syntelogs, with at least two retained duplicates in both *Amborella* – birch and birch – *Eucalyptus* comparisons, were enriched for the regulation of metabolic pathways, particularly nucleic acid metabolism and biosynthesis of macromolecules, alongside various stress responses (Table S8). Secondary cell wall cluster 9 genes (Fig. 4b; Tables S6, S8), which had retained synteny with *Amborella*, included 7% of the cluster genes and were enriched for xylan and pectin, as well as processes involved in modifying various CW components, such as glycosylation, galacturonosylation, and acetylation, and included a syntelog of the regulator MYB83. Conversely, birch syntelogs that had lost their syntenic relationship with *Amborella* and expanded in the gamma included lignin gene *PAL1*/*2*, *CCOAOMT1*, xylan *IRX15*/*15L*, *PARVUS*, and master regulators SND1‐NST1/2. While NAC domain genes have been found in land plants and green algae, their expansion occurred after the divergence of dicot plants and *Amborella* (Ohtani *et al*., 2017). This suggests that, in particular, the xylan and lignin gene families have expanded as a result of the gamma triplication.

We next analyzed the functional conservation of the syntelogs by comparing expression changes and regulatory regions. Syntelogs within the same cluster had a significantly higher median number of conserved motifs than those in different clusters (one‐sided Wilcoxon test, *P*‐value = 0.0153), suggesting hypofunctionalization or redundancy, as indicated by similar expression patterns and more conserved motifs (the same trend for SCW motifs only – Fig. S2; Table S9; Fig. S3 – Cluster 9 syntelog expression). However, out of 1614 syntenic duplicates, only 80 were identified in the same clusters, and overall, the cluster assignments among the top 10% of syntelogs with the highest proportion of conserved motifs showed great variation. These genes were enriched for response to stimulus, signaling, and macromolecule metabolism processes (Table S10), indicating candidates for sub‐ or neofunctionalization with conserved motifs but divergent expression.

We also explored the ratio of nonsynonymous to synonymous substitution rate (*K*_a_ : *K*_s_ ratio), which measures functional divergence through nonsynonymous substitutions (Table S11). Among the syntelogs, 121 had *K*_a_ : *K*_s_ > 1, indicating potential functional divergence and enrichment for regulation and signaling processes (Table S12). Notably, a duplicate of the SCW *Arabidopsis C4H* phenylpropanoid gene showed divergence with one duplicate in Cluster 9 and the other, excluded during CLUST clustering, peaked in F7. Similar *K*_a_ values for syntelogs within the same clusters (0.48) and across clusters (0.41) suggest comparable nonsynonymous substitution rates, with *K*_a_ : *K*_s_ ratios mostly below 1 (Table S11), indicating purifying selection. However, the observed average *K*_a_ values are not very low and could indicate relaxed purifying selection (Sandve *et al*., 2018), allowing for functional diversification to occur through some nonsynonymous mutation and/or expression changes.

### BpVND1/2/3 and BpNST1/2 are top‐layer regulators of SCW biosynthesis in birch, with high co‐expression conservation in poplar and *Eucalyptus*

The synteny analysis revealed fewer syntelogs in birch, *Eucalyptus*, and *Vitis* with *Amborella*, compared with poplar, indicating simpler regulatory interactions. Birch retained slightly more syntelogs with *Amborella* than *Eucalyptus*, suggesting greater conservation of the basal eudicot state (Fig. S1). We constructed a putative SCW gene regulatory network in birch and performed comparative transcriptomics to identify species‐specific regulatory interactions.

**Fig. 5:**
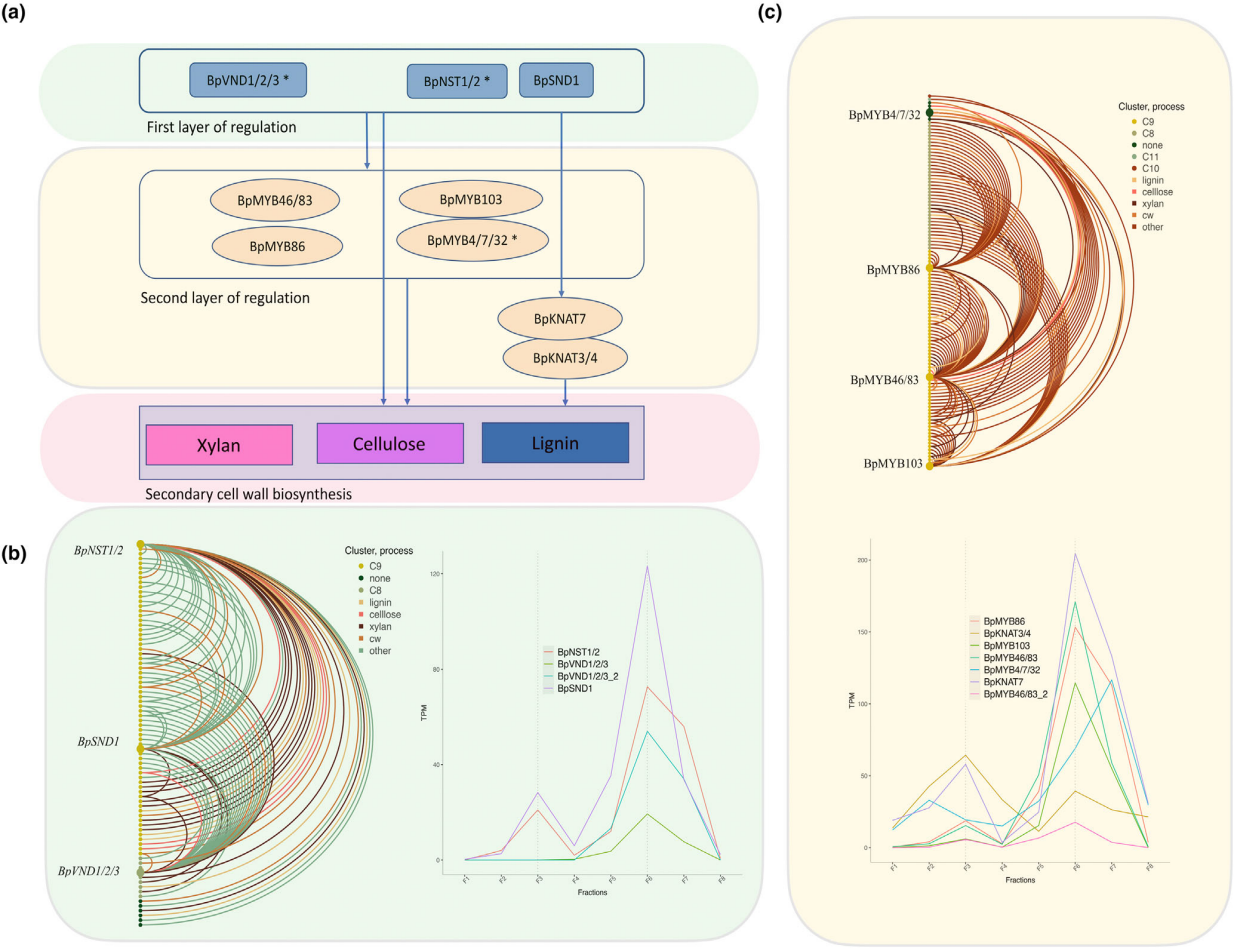
Cell wall biosynthesis regulatory network in birch. (a) Secondary cell wall biosynthesis regulators in Betula pendula. The percentage of conserved interactions is based on correlation (|*r*| > 0.7) and motif presence in Populus trichocarpa and Eucalyptus grandis. The percentages in brackets indicate conservation based on correlation alone. (b) First layer of regulation, including *BpNST1/2*, *BpSND1*, and *BpVND1/2/3*, along with their predicted downstream targets. Nodes are colored and sorted based on CLUST clusters, while edges are colored according to whether the Arabidopsis ortholog of the target gene is functionally associated with lignin, cellulose, or xylan biosynthesis, or other cell wall (CW)‐related processes. CW‐related processes include the biosynthesis of other cell wall polymers and cell wall organization. If a gene has two copies, only the one with the higher correlation to the target genes is shown in the network as the more likely regulator. (c) Second layer of regulation, including *BpMYB46/83*, *BpMYB103*, *BpMYB4/7/32*, and *BpMYB86*. Expression of *BpKNAT3/4* and *BpKNAT7* is also included as regulators based on linear regression analysis.

Motif enrichment and correlation analyses (Materials and Methods section, Tables S1, S13, S14) identified NAC and MYB family genes as regulators of the SCW clusters 8 and 9. The first layer of regulators (Figs 5a,b, S4; Table S15) included SCW master regulators BpNST1/2 (homolog to *Arabidopsis NST1* and *NST2*), *BpSND1*, and *BpVND1*/*2*/*3; BpSND1* was identified as a syntelog with *BpNST1*/*2*. By contrast, *Eucalyptus* retained two duplicates with sequence similarity to *SND1* genes, and no *NST*‐like genes. Poplar retained a comparable number of NAC regulators as *Arabidopsis* (Fig. S5). The targets of *BpSND1* and *BpVND1*/*2*/*3* were enriched for SCW biosynthesis GO categories, with fewer targets compared with *NST1*/*2* (Tables S15, S16; Fig. S4). Finally, *Arabidopsis VND1*‐*7* genes had only two homologs in birch: one for *VND1*‐*3* and one for VND7, with *VND4*‐*6* absent. *Eucalyptus* had two homologs of *VND4* and *VND*5, and one homolog for VND1, *VND2*, *VND3*, and *VND7*, similar to birch.

The top‐layer TFs regulate multiple downstream MYB TFs, including the homologs of second and third‐layer regulators MYB46/MYB83, BpMYB4/7/32, BpMYB86, and BpMYB103 (Table S15; Figs 5a,c, S6, S7). However, expression values of *BpMYB46/83* and downstream MYB TFs were not strongly correlated (*r* < 0.7). Downstream of BpNST1 and two HD‐ZIP TFs, BpHb30/34 and BpHB13/23, are suggested as regulators of several CW biosynthesis genes in the network (Table S15). While their homologs are not associated with CWs in *Arabidopsis*, HD‐ZIP class III genes, such as *REV*, *PHB*, and *PHAVOLUTA*, have known roles in xylem cell specification and regulation of lignin and cellulose biosynthesis genes (Du *et al*., 2011; Robischon *et al*., 2011; Taylor‐Teeples *et al*., 2015). We focused on the xylan biosynthesis genes and regulators in Notes S2 (Figs S8–S16).

Our results suggest that both *BpNST1*/*2* and *BpSND1* regulate second‐layer MYB genes, with *BpVND1*/*2*/*3* also acting as a regulator for BpMYB86. The bimodal expression of *SND*/*NST* genes suggests dual regulation of phloem and xylem fiber cells, whereas xylem‐specific expression of *VND* genes indicates preferential regulation in xylem vessels. The putative ortholog of *VND7* was only expressed in old xylem tissue and thus was not selected as a regulator. The network also includes multiple MYB TFs, such as BpMYB50/55/61 and BpMYB67, although their targets did not show enrichment for CW biosynthesis (Table S15).

In birch, as in *Arabidopsis* and poplar, BpMYB46 targets are involved in xylan, cellulose, and lignin biosynthesis (Kiim et al., 2013). While poplar has four MYB46/MYB83 homologs, birch has two, both with SCW‐specific expression. BpHB30/34 targets share enriched GO terms with those of BpMYB46/83. BpMYB103, similar to *Arabidopsis* MYB103 (Öhman *et al*., 2012), acts upstream of lignin and xylan biosynthesis. In *Arabidopsis* and poplar, MYB4, MYB7, and MYB32 negatively regulate SCW biosynthesis (Qin *et al*., 2020), and downstream of birch *BpMYB4*/*7*/*32* are genes that are orthologs of *Arabidopsis* MYB4/7/32 targets. Additionally, BpMYB86, like MYB86 in Arabidopsis, regulates lignin with targets enriched for CW biosynthesis genes.

The gene duplicates retained from the gamma polyploidization include xylan backbone *IRX14*/*14L*, *IRX15*, *IRX10*, *PARVUS*, *TBL* genes, *MYB83*, *HB30*/*34* in root growth, cellulose interactive *CSI* and *VND* interactive *VNI*, and lignin genes *CAD4*, *C4H*, *methyltransferase CCOAOMT1*, *LAC4*, *PAL1*/*2*, and *arabinogalactan methyltransferase*. Among them, the *PAL1*/*2* and multiple xylan genes have an *Amborella* syntelog suggesting preferential retention of lignin biosynthesis duplicates following the gamma event.

### Conservation and sensitivity analysis of SCW regulatory network

After constructing the CW network in birch, we assessed interaction robustness by varying the correlation thresholds between regulators and target genes and analyzing conservation in poplar and *Eucalyptus* (Table S15). When comparing birch with poplar, the conservation of interactions and TF binding motifs decreased from 61 to 24% as the correlation threshold was increased from 0.7 to 0.9. When comparing birch with *Eucalyptus*, there was a smaller drop from 47 to 34%, accompanied by a smaller overall percentage. Notably, in *Eucalyptus*, the presence of binding motifs 1 kb upstream of TSS had low recall, requiring analysis of the 2 kb upstream sequence for motif discovery.

Focusing on the conservation of regulatory interactions for NAC family master regulators, 71% and 58% downstream interactions of NST1/2 were conserved in poplar and *Eucalyptus*, respectively, while for VND1/2/3, 82% vs 57%. At the 0.9 threshold, conserved genes in *Eucalyptus* and birch were enriched for SCW biosynthesis, while in poplar, primarily for lignin biosynthesis. However, at the threshold of 0.7, xylan and cellulose targets were conserved by over 60%, and by 31% in lignin targets, in both species.

### Comparison with *P. trichocarpa* and *E. grandis* co‐expression modules suggests simplified regulatory architecture in birch and smaller gene families

We next used CLUST (Abu‐Jamous & Kelly, 2018) for multispecies clustering to identify conserved co‐expression modules. This method integrates ortholog information and gene expression data across species to identify clusters of orthogroups with consistently co‐expressed genes.

CLUST identified eight clusters of orthologs with conserved expression among all three species. Cluster 4, identified as an SCW cluster, contained 130 birch genes, with 26 and 19 genes from Clusters 9 and 8, respectively, from the birch‐specific analysis (Fig. S17). The corresponding poplar cluster had 296 genes, while *Eucalyptus* had 365. The orthogroups shared across all species were associated with cellulose, xylan, pectin biosynthesis, and cell growth processes. The cluster had four birch‐specific orthogroups, while poplar and *Eucalyptus* had 30 and 39, respectively (Table S17). Birch‐specific orthogroups were enriched for CW organization, while *Eucalyptus*‐specific ones were driven by tandem duplications and were linked to water homeostasis and phenylpropanoid regulation. Poplar‐specific orthogroups showed no specific enrichments.

Separate CLUST analyses in poplar and *Eucalyptus* allowed species‐specific adjustment of the clustering tightness parameter to account for differences in gene counts. CLUST identified 12 clusters in poplar, with Clusters 4 and 5 enriched for SCW (Fig. S18; Table S18). Cluster 9 showed enrichments for phloem and xylem histogenesis, and Cluster 12 for CW organization and modification. The orthologs related to SCW cellulose synthesis were in Cluster 4, while PCW CesA genes were spread across Clusters 2, 6, and 12. Lignin biosynthesis genes were found in Clusters 4 and 5, and some were omitted during clustering. Cluster 5 exhibited coordinated expression of regulators KNAT7, MYB20, MYB42/85, MYB52/54, MYB83, and MYB103, contributing to lignin biosynthesis in *Arabidopsis* (McCarthy *et al*., 2010; Li *et al*., 2015). Out of these, birch network analyses highlighted BpMYB42/85, in addition to MYB103 and KNAT7, as a potential regulator of *BpC4H* and *Bp4CL1*, emphasizing fewer lignin regulators in birch.

In *Eucalyptus*, 15 clusters were identified, with Cluster 13 enriched for pectin biosynthesis. Lignin biosynthesis genes clustered predominantly in Cluster 1 (2711 genes). However, this cluster showed enrichment for other processes, such as jasmonic acid signaling and peptide biosynthesis. Xylan genes were found in multiple clusters without enrichment for lignin‐related terms (*t* = 5, Cluster 11 enriched for CW and xylan).

### KNAT7 is a putative regulator of lignin biosynthesis

Even though KNAT7 is a well‐recognized regulator of SCW biosynthesis, its binding site remains unknown, preventing us from applying the same criteria for the network construction. More specifically, KNAT7 is a direct regulator of xylan biosynthesis and monolignol, and it can act in concert with *KNAT3* either as a negative or positive regulator (He *et al*., 2018; Qin *et al*., 2020). We used linear regression to identify potential targets of the KNOX II TFs. Target gene expression was predicted using *KNAT7* and *KNAT3* as covariates, having either positive and/or negative regulatory roles (Figs S19, S20). The likelihood ratio test was subsequently used to select the best linear model among the different candidates, and potential targets for which at least 70% of the variance was explained with the model were selected. The findings suggested *KNAT7* as a positive regulator of lignin biosynthesis together with *BpKNAT3*, as recently indicated for monolignol biosynthesis in *Arabidopsis* (Qin *et al*., 2020; Tables S19–S22; Notes S3).

### Xylan PCW biosynthesis genes show distinct expression patterns and lack the common PCW regulatory motifs

The PCWs, deposited during cell division and elongation, remain less understood in terms of regulation (Bosca *et al*., 2006; Moneo‐Sánchez *et al*., 2020). Unlike SCWs, PCWs are nonlignified, and engineering plants with PCWs instead of SCWs could reduce recalcitrance, improving biomass extraction for industrial applications (Sakamoto *et al*., 2018). We therefore analyzed xylan PCW biosynthesis genes, focusing on Cluster 16 (Fig. 3), enriched for cellulose, glucan, pectin, and xyloglucan synthesis, alongside their expression patterns and regulation (Fig. S21). In addition to this, we analyzed the monomeric sugar concentrations and the correlation of PCW vs. SCW sugars and gene expression (Notes S4; Figs S22–S25).

Only four *Arabidopsis* genes are associated with xylan biosynthesis in PCWs (Mortimer *et al*., 2015). Recently, a gene within the subfamily of GT47‐A Golgi glycosyltransferases was identified as xylan arabinopyranosyltransferase (*XAPT1*) in *Arabidopsis*, responsible for attaching arabinopyranose to xylan glucuronic acid. In *Myrtaceae*, neofunctionalization has led to xylan galactosyltransferases that decorate MeGlcA with galactose (Yu *et al*., 2024). In our analyses, homologs of these genes showed unique expression patterns, differing from both SCW and other PCW biosynthesis genes (Notes S2). For instance, the expression of the glucuronylation ortholog of *GUX3* peaked in cork cambial tissues, while other genes were active in the vascular cambium where PCW biosynthesis is initiated (Fig. S21). The homolog of *Arabidopsis* SCW acetylation gene *BpTBL35* showed similar expression to *GUX3*, indicating a potential role in PCW biosynthesis in birch (Fig. S26). Another gene with a putative function in PCW biosynthesis was found in the same orthogroup as *BpIRX9L*, with expression similar to *BpIRX9L* (Notes S2).

The RCCGAC binding motif for the ethylene response factor (ERF) subfamily of AP2 transcription factor genes, regulators of cellulose biosynthesis in *Arabidopsis* PCW (Sakamoto *et al*., 2018; Saelim *et al*., 2019), was found upstream of PCW cellulose synthases and xyloglucan genes but not xylan PCW genes. Instead, xylan *GUX3* contained motifs for homeobox (HB), squamosa, C2H2 BIRD, DNA‐binding One Zinc Finger (DOF) proteins, and WRKY29/40, indicating diverse regulatory processes warranting further study.

### Distinct MeGlcA‐*O*‐acetyl‐POS profiles in young phloem and xylem

Xylan modifications are crucial for interactions with cellulose and lignin, affecting the CW composition and strength. However, their regulation in woody plants is not well understood (Hao & Mohnen, 2014; Pereira *et al*., 2017). Additionally, xylan structures in the literature often represent averages across all SCW tissues, masking spatial heterogeneity within tree stems. To address this, we analyzed the composition and content of xylan in birch fractions at tissue‐specific resolution (Chong *et al*., 2014) and linked these findings to the gene expression profiles. In addition, we analyzed the noncellulosic sugar concentrations with acid methanolysis and gas chromatography (Note S4).

**Fig. 6:**
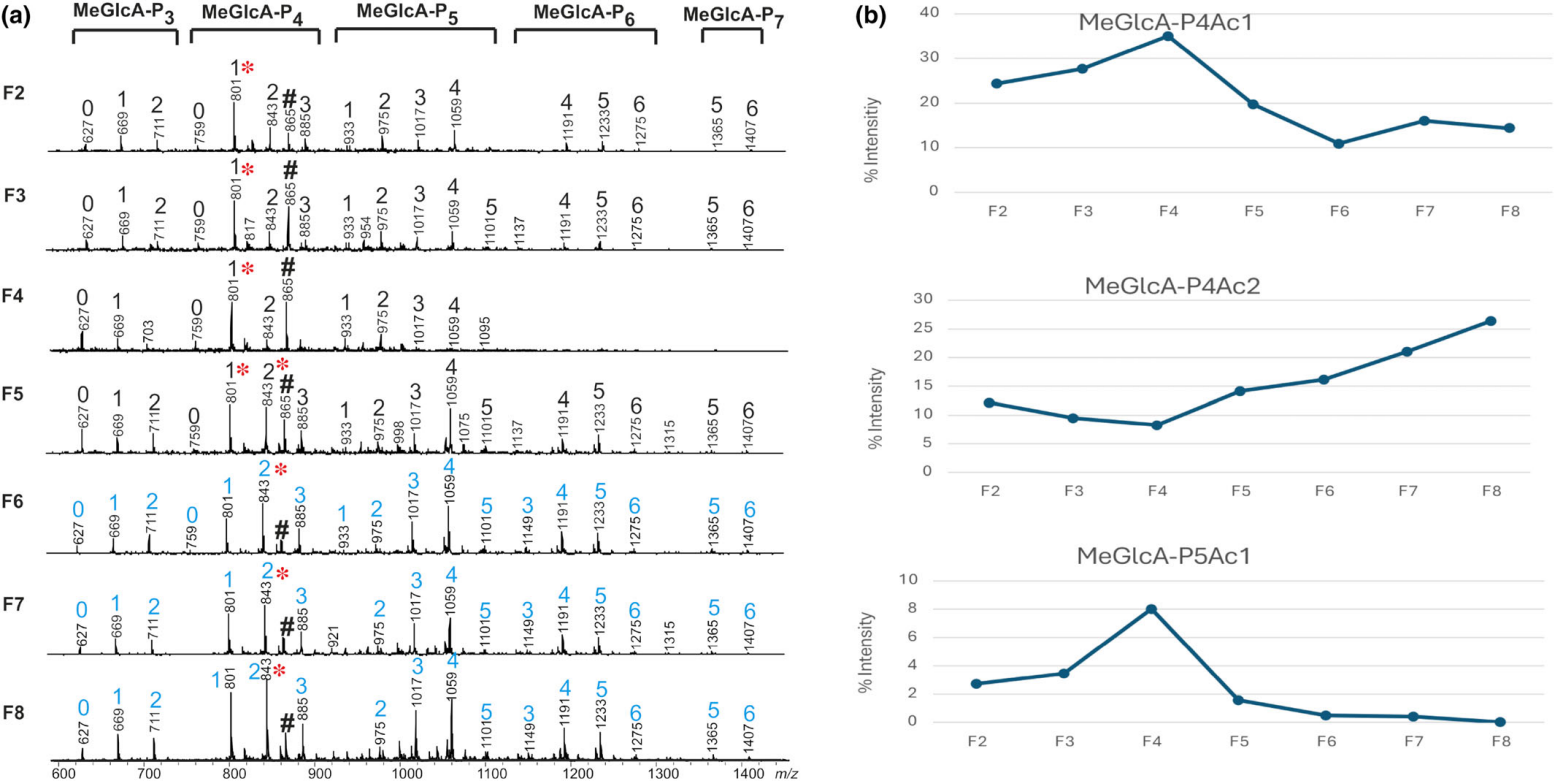
Alcohol‐insoluble residues of birch tissue‐specific wood fractions were hydrolyzed by AaGH10 endoxylanase and analyzed with atmospheric pressure‐matrix assisted laser desorption/ionization (AP‐MALDI)‐ion trap mass spectrometry (ITMS). (a) Mass spectra for MeGlcA‐O‐acetyl‐pentosyl oligosaccharide. The number above the peak indicates the number of acetyl groups, * marks the main peak(s); and No. of the internal standard. (b) Mass list was exported, and the intensity ratio for the selected peak to that of the internal standard was calculated and summed up as 100%. Peaks associated with xylem P4Ac2, and peaks associated with developing phloem P4Ac1 and P5Ac1.

We found distinct MeGlcA‐O‐acetyl‐pentosyl oligosaccharide (acidic POS) profiles for phloem and xylem, with young phloem exhibiting the most distinct modification profile (Figs 6a, S27). Acidic POS from young (F6) and old (F7 and F8) xylem showed three to seven pentosyl residues (MeGlcA‐P3‐7) with one to six acetyl residues, peaking at MeGlcA‐P4Ac2, consistent with previous hardwood stem findings (Chong *et al*., 2014). By contrast, young phloem's acidic POS was shorter and lacked an acetyl substitution on the MeGlcA‐linked xylose residue. The vascular cambium combined young phloem and xylem profiles, indicating ongoing cell division and differentiation.

The xylan in young phloem possessed a tight MeGlcA substitution cluster with one MeGlcA residing in every six xylose residues, as opposed to one MeGlcA in every eight or ten xylose residues in the xylem. Tandem MS (MS^n^) analysis of the phloem‐specific peak (Fig. S28) demonstrated that it lacked one acetyl substitution on the MeGlcA‐linked xylose residue compared with the xylem peak. The single acetyl substitution was found on the second pentose residue from the reducing end (Notes S5, S6). The young phloem peak resulted in shorter fragments than those of xylem, with the longest acidic POS comprised of five pentoses that were either non‐ or monoacetylated. Both peaks, MeGlcA‐P_5_ and MeGlcA‐P_5_Ac (Fig. S29), were further analyzed to examine the position of the substitutions. The analysis suggested the occurrence of a pentose‐MeGlcA disaccharide side branch located at the nonreducing end of the xylotetraose (Notes S7). Analysis of the *Bo*GH30 hydrolyzed deacetylated phloem xylan with carbohydrate gel electrophoresis suggested xylooligosaccharide harboring L‐Ara*p*‐MeGlcA disaccharide side branch (Notes S8, S9; Figs S30–S32) previously assigned as a putative α(1→2)‐linked l‐arabinopyranose (Ara*p*) in *Arabidopsis* (Chong *et al*., 2014; Mortimer *et al*., 2015) and recently in *Eucalyptus* (Yu *et al*., 2024). The NMR spectroscopic analysis of deacetylated xylan further supported that in the side branch, α‐Ara*p* was (1→2) linked to a α‐MeGlc*p*A residue (Notes S5; Table S23; Fig. S33). Compared with young xylem, the young phloem xylans contained less mono‐ and di‐acetylation mainly attributed to reduction in 2‐*O*‐acetyl‐Xyl (X2) and 3‐*O*‐acetyl‐Xyl (X3), as well as 2,3‐*O*‐acetyl‐Xyl (X23) (Tables S23, S24).

To identify potential arabinopyranosyltransferase in birch, we used the *Arabidopsis* XAPT1 sequence to generate a phylogenetic tree with MAFFT and TBLASTN results from birch, poplar, *Amborella*, and *Eucalyptus* (Fig. S34). The closest homologs of *Arabidopsis* and *Eucalyptus* genes were not expressed in birch, nor were the poplar genes in the same clade (Sundell *et al*., 2017). Since we observed the l‐arabinopyranose (Arap) structure in young phloem, but no corresponding expression, further quantification of expression is needed. Alternatively, functional divergence might have progressed along different trajectories in birch and poplar, and a related PCW glucosyltransferase might be responsible for this modification.

Given the distinct acetylation patterns observed in PCW tissues, we performed correlation analyses to link xylan structure, as reflected by the main peaks of xylan oligosaccharides in PCW and SCW tissues, with gene expression, aiming to identify potential acetylation genes (Figs 6, S28; Table S25). While SCW xylan biosynthesis genes exhibited SCW‐specific patterns, the main peak intensity profile in the xylem strongly correlated with the tissue‐wide expression of *BpXND1*, a NAC domain TF. *BpXND1* is known to regulate SCW deposition in *Arabidopsis* by inhibiting *VND6* and *SND1* expression (Zhang *et al*., 2018; Zhong *et al*., 2021). Other top‐correlated genes include those involved in xylem differentiation and vascular development (homologs of ACL5, HB8, MYBS2, and WRKY12), as well as MAP70‐5, involved in SCW patterning in *Arabidopsis*. The list also includes MYB52 and MYB54 homologs, which regulate SCW thickening, CW‐associated kinases, and xylem development genes (Tables S26, S27; Figs S35, S36) The fact that the most abundant xylan structure in the xylem correlates with genes controlling the activity of the SCW master regulators could suggest a role of the metabolite concentrations on the biosynthesis and deposition of the CW, as well as the interaction with vascular development through a potential metabolite‐transcriptome feedback.

In young phloem, the main peak related to PCW correlates with genes enriched for organic substance synthesis, including xyloglucan and lignin biosynthesis genes. The homolog of the acetylation gene *TBL27* appeared as a candidate for PCW acetylation (Tables S26, S27; Fig. S37).

## Discussion

Here, we conducted a comprehensive analysis of RNA‐sequencing data from bark to xylem tissues in birch, focusing on both PCW and SCW regulatory networks. We identified co‐expression modules of PCW and SCW biosynthesis and integrated TF binding motif analysis to construct a transcriptional regulatory network of SCW. We obtained key insights into the regulatory architecture of CW biosynthesis, namely the identification of *BpNST/SND* as master regulators in fiber phloem and xylem cells and *VND* as regulators specifically expressed in xylem cells, and their downstream *MYB* regulators and target genes. Network analysis suggested conserved interactions between the first‐layer regulators *BpNST1/2* and *BpVND1/2/3* and their targets in poplar and birch, with species‐specific clustering indicating more regulators in poplar. Genes originating from WGM were enriched among the SCW clusters, indicating their preferential retention and relevance of SCW biosynthesis in providing adaptive capabilities with improved stress resilience and nutrient transport (Sørensen *et al*., 2012; Takata & Taniguchi, 2015). Additionally, tissue‐specific wood fractions showed lower MeGlcA substitution in xylans of mature xylem compared with other tissues, with distinct acetylation patterns in vascular cambium and young phloem tissues.

The distinct expression pattern of PCW xylan biosynthesis, with occasional peaks in cork cambium (F2), suggests unique regulation compared with other PCW components. Multiple lignification genes, including homologs of *MED5A, PRX25*, and *PDR1*, as well as one paralog of *4CL1* and *PRX71*, also peaked in F2. The bark contains significant amounts of lignin, with 22–29 wt% in species, such as poplar, oak, and pine (Vangeel *et al*., 2021). The expression of these genes in the cork cambium potentially contributes to CW formation in birch bark tissues, since, for example, in *Quercus suber*, SCW genes were found active during late cork formation, with a suggested role in CW thickening (Fernández‐Piñán *et al*., 2021).

A basal angiosperm, such as *Amborella trichopoda*, offers a useful reference to identifying changes that occurred during angiosperm diversification. Since the *Betula pendula* lineage has undergone only the gamma triplication since its split with the *Amborella* lineage, genome comparisons with *Amborella* helped us assess the gene retention and the added functional complexity subsequent to the gamma event. Less than 20% of birch genes retained their gamma duplicates, with only a few involved in the SCW network. Over 125 million years of evolution, dosage sensitivity and functional divergence have shaped duplicate retention, while diploidization has favored a return to a single‐copy genome state. Redundant regulators with overlapping functions, especially in CW biosynthesis, may be lost over time. However, even with the loss of some regulators, retained downstream target genes can help maintain dosage balance in the CW machinery. Duplicates, such as *MYB46* and *MYB83*, contribute to redundancy in poplar and *Arabidopsis* (McCarthy *et al*., 2009, 2010); their syntelogs were present in birch as well, but one duplicate demonstrated significantly lower expression levels than the other. On the other hand, the SCW co‐expression module was enriched for gamma syntelogs, and among these duplicates, only two xylan genes retained synteny with *Amborella*. Analysis of the *Amborella* genome suggested that orthogroups containing vessel formation genes, such as *VND7* first appeared in angiosperms (Amborella Genome Project, 2013). *Amborella* does not produce vessels but instead has tracheary cells in its xylem, suggesting that genes involved in CW synthesis and deposition in vessels might not have served this purpose in its genome. The lignin composition in *Amborella* resembles that of gymnosperms, and while the xylan content is similar, many CW genes evolved later after the divergence from *Amborella*, and the SCW syntelogs could originate from the tetraploid ancestor contributing to the gamma hybridization event.

Overall, birch appears closest to the ancestral eudicot state, with more retained syntelogs compared with *Amborella*, while *Eucalyptus* had more tandemly expanded genes. We identified an SCW network in birch and assessed the conservation of interactions in poplar and *Eucalyptus*. Top‐layer regulators showed high conservation compared with second‐layer MYB TFs. We observed fewer duplicates of the *VND* genes in birch, with a potential role in xylem regulation due to a unimodal expression peak. Similarly, in *Eucalyptus*, a set of woody species‐specific MYBs demonstrated expression peaks in the cambium, suggesting a role in the regulation of secondary development (Soler *et al*., 2015). Both cases can be considered as species‐specific regulation of the top‐layer regulators. The overall level of conservation of the regulatory network was higher in poplar than in *Eucalyptus*. This could be due to lower tissue resolution in RNA‐sequencing data; unlike birch and poplar, *Eucalyptus* samples lacked old phloem and had fewer xylem replicates (Vining *et al*., 2015; Sundell *et al*., 2017; Alonso‐Serra *et al*., 2019). Differences in tissue sampling resolution between poplar and birch further limited direct comparisons of conserved regulation.

While birch lacks a WGM after the gamma, *Eucalyptus* contains the Myrtales duplication with many tandem duplicates (37%), while the Salicaceae ancestor of poplar experienced a duplication 58 Ma. In both species, the events contributed to gene family expansions and possibly to divergent regulation. Multispecies clustering revealed little overlap between multiclust and single‐species clusters, likely associated with methodological difficulties, as the current models cannot take into account the WGDs and varying gene numbers in plant species. Xylan and pectin expression was more conserved across the three species, with poplar and *Eucalyptus*‐specific orthogroups, including more genes involved in phenylpropanoid biosynthesis, and the genes in the *Eucalyptus*‐specific orthogroups containing tandemly expanded duplicates. Together, this suggests that lignin families may have expanded through WGM in poplar and tandem duplications in *Eucalyptus*.

Our analyses demonstrated that xylan structures were spatially dispersed – reflecting the structural differences between PCW and SCW in woody tissues. Taken together, the unusual acetyl and MeGlcA substitutions in primary wall xylans suggest a different functional role from those present in SCW, where the xylans have higher acetylation levels and complex MeGlcA substitution patterns (Bromley *et al*., 2013; Busse‐Wicher *et al*., 2014; Chong *et al*., 2014). The regular spacing of both acetyl and MeGlcA substituents in SCW xylan dictates the folding of xylan onto cellulose (Simmons *et al*., 2016), which may affect the rigidity of CW and mechanical support in plants. The unusual acetyl and MeGlcA substitutions, together with the unique Ara*p*‐MeGlc*p*A side branch in xylan, are likely intended for gluing the wall components for extensibility and plasticity of the primary wall. Our study uncovers details on the substitution pattern in xylans that will benefit the study of the physicochemical properties of the PCW in woody tissues.

## Competing interests

None declared.

## Author contributions

JS and MT planned the study. S‐LC and MT conducted the chemical and molecular profiling of xylan. JW‐R and PD contributed to the carbohydrate gel electrophoresis (PACE) analysis. HM contributed to the NMR data acquisition. MI performed the computational analysis and implementations. JI and KN contributed the birch material and sectioning of the stem for chemical studies. YH provided the material and input for the manuscript. MI, S‐LC and JS wrote the manuscript with input from JO, K‐JL and MT. All authors read and approved the final manuscript. MI and S‐LC contributed equally to this work.

## Disclaimer

The New Phytologist Foundation remains neutral with regard to jurisdictional claims in maps and in any institutional affiliations.

## Supporting information


**Fig. S1** Syntelog copy numbers, percentages and tandem duplications.
**Fig. S2** Conserved SCW motifs among syntelogs vs Pearson correlation.
**Fig. S3** Expression (TPM) pattern of the syntenic duplicates in Cluster 9.
**Fig. S4** Cell wall biosynthesis regulatory network in birch, first layer.
**Fig. S5** Gene tree of the NAC transcription factors orthogroup.
**Fig. S6** Cell wall biosynthesis regulatory network in birch, second layer.
**Fig. S7** Gene tree of the second‐layer MYB transcription factors orthogroup.
**Fig. S8** Gene tree of the xylan backbone genes IRX10, IRX10L.
**Fig. S9** Phylogenetic tree of the xylan backbone gene IRX9 orthogroup.
**Fig. S10** Phylogenetic tree of the xylan backbone gene IRX9L orthogroup.
**Fig. S11** Expression of the *BpIRX9L* and a potential paralog from the same orthogroup.
**Fig. S12** Phylogenetic tree of the xylan backbone genes *IRX14, IRX14L* orthogroup.
**Fig. S13** Expression of the *BpIRX14/14L* and a potential paralog from the same orthogroup.
**Fig. S14** Gene tree of the xylan glucuronylation genes, GXM orthogroup.
**Fig. S15** Phylogenetic tree of the xylan acetylation genes RWA orthogroup.
**Fig. S16** Xylan biosynthesis in *Betula pendula* and predicted regulators.
**Fig. S17** GO BP enrichment of the orthogroups in the SCW cluster 4, based on clustering of gene expression datasets from multiple species, with CLUST.
**Fig. S18** *Populus trichocarpa*, expression profiles of the genes for each cluster.
**Fig. S19** Expression (TPM) profile of *BpKNAT7* and *BpKNAT3*.
**Fig. S20** GO biological processes and molecular function enrichment of the genes predicted to be co‐regulated by *BpKNAT7* and *BpKNAT3*.
**Fig. S21** Expression (TPM) of the PCW biosynthesis genes in the birch orthologs.
**Fig. S22** Monomeric sugar content (% dry weight) across the stem fractions.
**Fig. S23** PCA of the monomeric sugar content.
**Fig. S24** Pearson correlation of the clusters mean expression and the sugar content (% dry weight).
**Fig. S25** PCA of transformed sugar profiles and Pearson correlation.
**Fig. S26** Expression (TPM) of the orthologs of the Arabidopsis TBL gene family, involved in acetylation of cell wall.
**Fig. S27** Xylan oligosaccharide mass profiling analysis of developmental tissues in birch wood.
**Fig. S28** Structural study of the main peak (MeGlcA‐P_4_Ac) present in the acidic fraction of AaGH10 endoxylanase hydrolysate from young phloem.
**Fig. S29** Structural study of the minor peak MeGlcA‐P_5_Ac present in the acidic fraction of *Aa*GH10 endoxylanase hydrolysate from young phloem.
**Fig. S30** MeGlcA substitution pattern of birch young xylem and phloem xylan.
**Fig. S31** Comparison of qHSQC spectra of TrGH11 hydrolyzed O‐acetylglucuronoxylans isolated from young xylem (red) and young phloem (black).
**Fig. S32** qHSQC spectrum obtained from the TrGH11 hydrolyzed deacetylated glucuronoxylans isolated from young phloem.
**Fig. S33** Nuclear Overhauser Effect Spectroscopy (NOESY) spectrum obtained from deacetylated glucuronoxylans isolated from young phloem.
**Fig. S34** Gene tree of XAPT‐like sequences.
**Fig. S35** Expression of the genes that correlate (absolute correlation > 0.7) with the P4Ac2 xylem MS peak.
**Fig. S36** Expression of the orthologs of xylan SCW biosynthesis genes in *Betula pendula*.
**Fig. S37** Expression profile of the genes that correlate with the phloem MS peaks.
**Notes S1** Weighted Gene Co‐expression Network Analysis (WGCNA) clustering.
**Notes S2** Xylan biosynthesis in *Betula pendula* and regulation of cell wall biosynthesis.
**Notes S3** KNAT7 is a putative regulator of lignin biosynthesis.
**Notes S4** Xylan concentration, monomeric sugar composition.
**Notes S5** Xylan MS analysis.
**Notes S6** The main peak in hydrolysate of young phloem lacked acetyl substitution in the MeGlcA‐linked xylose residue.
**Notes S7** The disaccharide side branch pentose‐MeGlcA was detected in young phloem xylans.
**Notes S8** PACE analysis revealed primary wall‐like MeGlcA substitution pattern and Ara‐MeGlcA side branch in young phloem xylans.
**Notes S9** Two‐dimensional NMR analysis confirmed lower acetylation level and an α‐Ara*p*(1→2)‐α‐MeGlc*p*A side branch in the xylans from young phloem.

**Table S1** Significantly enriched GO terms (Biological process) for the CLUST clusters based on enrichment analysis with goatools.
**Table S2** Gene Ontology (GO) BP enrichment of the WGCNA Clusters.
**Table S3** Fisher's exact test for overlap between CLUST and WGCNA clusters.
**Table S4** Cell wall biosynthesis genes in birch.
**Table S5** Module membership (MM) and degree of connectivity from WGCNA analysis for each gene included in the CLUST clustering.
**Table S6** Fisher exact test for overrepresentation of duplicates originating from whole‐genome duplications (syntenic) or tandem duplication among the gene sets associated with cell wall biosynthesis or CLUST clusters.
**Table S7** Syntenic duplicates among the clusters that are enriched for whole‐genome duplication copies, cl9, cl11, cl16.
**Table S8** GO enrichment of the birch genes with 3 preserved copies from whole‐genome duplication based on syntenic alignment of the birch and Amborella genomes and genes with 2 copies in eucalyptus based on birch‐eucalyptus syntenic alignment.
**Table S9** Birch genes involved in cell wall (CW) biosynthesis and originating from whole‐genome or tandem duplications.
**Table S10** GO enrichment of the top 10% of the syntelogs based on the number of conserved binding motifs 1 K upstream transcription start site (TSS).
**Table S11** Syntelogs in birch detected through self‐alignment.
**Table S12** GO enrichment of the syntelogs with nonsynonymous substitution rate (*K*_a_) over 1.
**Table S13** Promoter motif enrichment of the birch clusters with Fisher exact test.
**Table S14** Enriched motifs in the cell wall clusters, and correlation between the cluster target gene and the transcription factor with the binding motif.
**Table S15** SCW biosynthesis network, combining promoter motif enrichment and correlation.
**Table S16** BP GO enrichment of the targets of the SCW regulators. GO terms with Bonferroni corrected *P*‐value < 0.05 are reported.
**Table S17** Genes and orthogroups (OG) in Cluster 4 of the multispecies clustering (birch, poplar and eucalyptus), enriched for CW biosynthesis.
**Table S18** GO enrichment of the poplar clusters.
**Table S19** Results from the linear regression analysis using the model lm(response ~ knat7 × knat3), with BpKNAT3 and BpKNAT7 as covariates.
**Table S20** GO enrichment of the genes explained by the model lm(response ~ knat7 × knat3).
**Table S21** Results from the linear regression analysis using the model lm(response ~ knat7 + knat3), with BpKNAT3 and BpKNAT7 as covariates.
**Table S22** Genes for which the reduced model lm(response ~ knat7) is significant and the ANOVA model testing rejects the full model lm(response ~ knat7 + knat3).
**Table S23** 1H assignment of deacetylated xylans from young phloem.
**Table S24** Relative content (%) of substituted Xylp in the O‐acetylglucuronoxylans from young phloem (F4) and xylem (F6).
**Table S25** Percentage intensity ratio of the mass spectrometry peaks (intensity ratio of selected peak to that of internal standard).
**Table S26** Correlation between the gene expression and the mass spectrometry peak intensity ratios.
**Table S27** GO enrichment of the genes correlating with the mass spectrometry peaks with goatools.
Please note: Wiley is not responsible for the content or functionality of any Supporting Information supplied by the authors. Any queries (other than missing material) should be directed to the *New Phytologist* Central Office.

## Data Availability

All sequencing data have been deposited in the European Nucleotide Archive under the accession code: PRJEB29260.
