# Supplementary material for: Gene regulatory network analysis of silver birch reveals the ancestral state of secondary cell wall biosynthesis in core eudicots
Source: New Phytol. 2025 Apr 16;246(5):2059–74. doi: 10.1111/nph.70126 (PMC12059548; doi:10.1111/nph.70126)
Supplement: Supplementary file 1 — Fig. S1 Syntelog copy numbers, percentages and tandem duplications. Fig. S2 Conserved SCW motifs among syntelogs vs Pearson correlation. Fig. S3 Expression (TPM) pattern of the syntenic duplicates in Cluster 9. Fig. S4 Cell wall biosynthesis regulatory network in birch, first layer. Fig. S5 Gene tree of the NAC transcription factors orthogroup. Fig. S6 Cell wall biosynthesis regulatory network in birch, second layer. Fig. S7 Gene tree of the second‐layer MYB transcription factors orthogroup. Fig. S8 Gene tree of the xylan backbone genes IRX10, IRX10L. Fig. S9 Phylogenetic tree of the xylan backbone gene IRX9 orthogroup. Fig. S10 Phylogenetic tree of the xylan backbone gene IRX9L orthogroup. Fig. S11 Expression of the BpIRX9L and a potential paralog from the same orthogroup. Fig. S12 Phylogenetic tree of the xylan backbone genes IRX14, IRX14L orthogroup. Fig. S13 Expression of the BpIRX14/14L and a potential paralog from the same orthogroup. Fig. S14 Gene tree of the xylan glucuronylation genes, GXM orthogroup. Fig. S15 Phylogenetic tree of the xylan acetylation genes RWA orthogroup. Fig. S16 Xylan biosynthesis in Betula pendula and predicted regulators. Fig. S17 GO BP enrichment of the orthogroups in the SCW cluster 4, based on clustering of gene expression datasets from multiple species, with CLUST. Fig. S18 Populus trichocarpa, expression profiles of the genes for each cluster. Fig. S19 Expression (TPM) profile of BpKNAT7 and BpKNAT3. Fig. S20 GO biological processes and molecular function enrichment of the genes predicted to be co‐regulated by BpKNAT7 and BpKNAT3. Fig. S21 Expression (TPM) of the PCW biosynthesis genes in the birch orthologs. Fig. S22 Monomeric sugar content (% dry weight) across the stem fractions. Fig. S23 PCA of the monomeric sugar content. Fig. S24 Pearson correlation of the clusters mean expression and the sugar content (% dry weight). Fig. S25 PCA of transformed sugar profiles and Pearson correlation. Fig. S26 Expression (TPM) of the orthol [file NPH-246-2059-s001.docx]

## *New Phytologist* Supporting Information

Article title: **Gene regulatory network analysis of silver birch reveals the ancestral state of secondary cell wall biosynthesis in core eudicots**

Authors: Maja Ilievska, Sun-Li Chong, Kean-Jin Lim, Juha Immanen, Kaisa Nieminen, Hannu Maaheimo, Ykä Helariutta, Joel Wurman-Rodrich, Paul Dupree, James Ord^,^ Maija Tenkanen, Jarkko Salojärvi

Article acceptance date: 12 March 2025

The following Supporting Information is available for this article:

**Fig. S1** Syntelog copy numbers, percentages and tandem duplications.

Syntelog copy numbers and percentages in **(a)** *A. trichopoda* vs *B. pendula*, *P. trichocarpa*, *A.thaliana*, *E.grandis* and *V.Vinifera*. 1:1 synteny (one-to-one) indicates that a single gene in *A. trichopoda* corresponds to a single gene in one of the other species as a syntelog; 1:2 synteny means that one gene in *A. trichopoda* has two syntelogs in the other species, and so on.  **(b)** Number of tandem duplications for each species.

**Fig. S2** Conserved SCW motifs among syntelogs vs Pearson correlation.

Conserved SCW motifs vs Pearson correlation coefficient for syntelogs within the same cluster or different clusters (including non-clustered syntelogs). Horizontal line inside the box – median value; the box - interquartile range (IQR), showing the 25th–75th percentiles; **whiskers** - the upper and lower whiskers represent scores outside the middle 50% (i.e., the lower 25% of scores and the upper 25% of scores); outliers – points outside the whiskers indicating potential extreme values.

**Fig. S3** Expression (TPM) pattern of the syntenic duplicates in cluster 9.

Expression (TPM) pattern of the syntenic duplicates in cluster 9. For each syntelog pair at least one of the copies is in cluster 9.

**Fig. S4** Cell wall biosynthesis regulatory network in birch, first layer.

Cell wall biosynthesis regulatory network in birch. First layer of regulation including *BpNST1/2, BpSND1 and BPVND1/2/3* as regulators and their predicted downstream targets*.* The nodes are sorted and colored based on the CLUST cluster, while the edges are colored based on whether the Arabidopsis ortholog of the target gene is functionally related to biosynthesis of lignin, cellulose, xylan cell wall (cw) or other processes. CW related processes include biosynthesis of other polymers in the cell wall and cell wall organization. Genes are named with the” Bp” prefix and the name of the Arabidopsis homolog.

**Fig. S5** Gene tree of the NAC transcription factors orthogroup.

Gene tree of the NAC transcription factors orthogroup inferred with Orthofinder. Protein sequences were aligned with MAFFT and gene tree was estimated with FastTree.


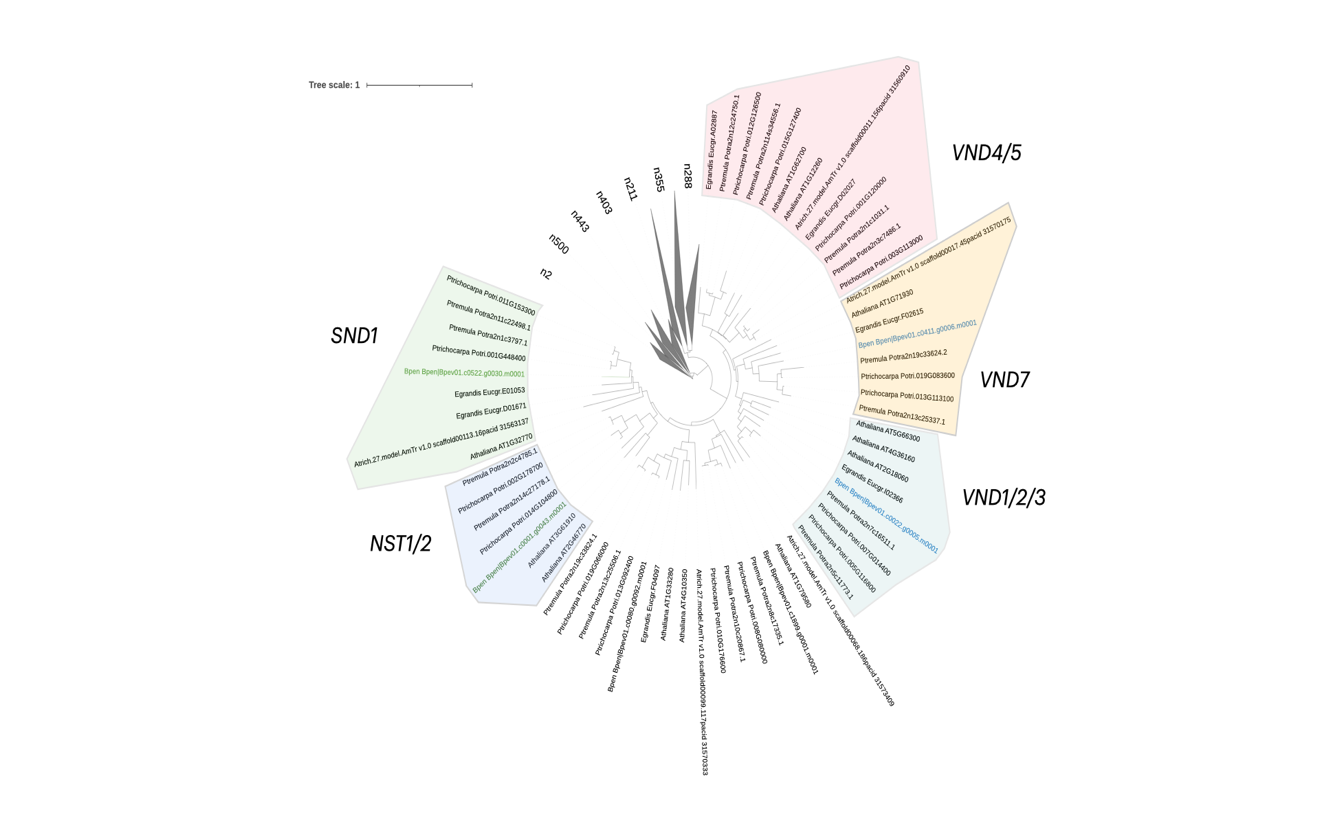


**Fig. S6** Cell wall biosynthesis regulatory network in birch, second layer.

Cell wall biosynthesis regulatory network in birch, second and third layer. Second layer regulators BpMYB46/83 and third layer BpMYB103, BpMYB4/7/32 and BpMYB86. The nodes are sorted and colored based on the CLUST cluster, while the edges are colored based on whether the closest Arabidopsis homolog of the target gene is functionally related to biosynthesis of lignin, cellulose, xylan, cell wall (cw). CW related processes include biosynthesis of other polymers in the cell wall and cell wall organization. Genes are named with the ”Bp” prefix and the name of the Arabidopsis homolog.


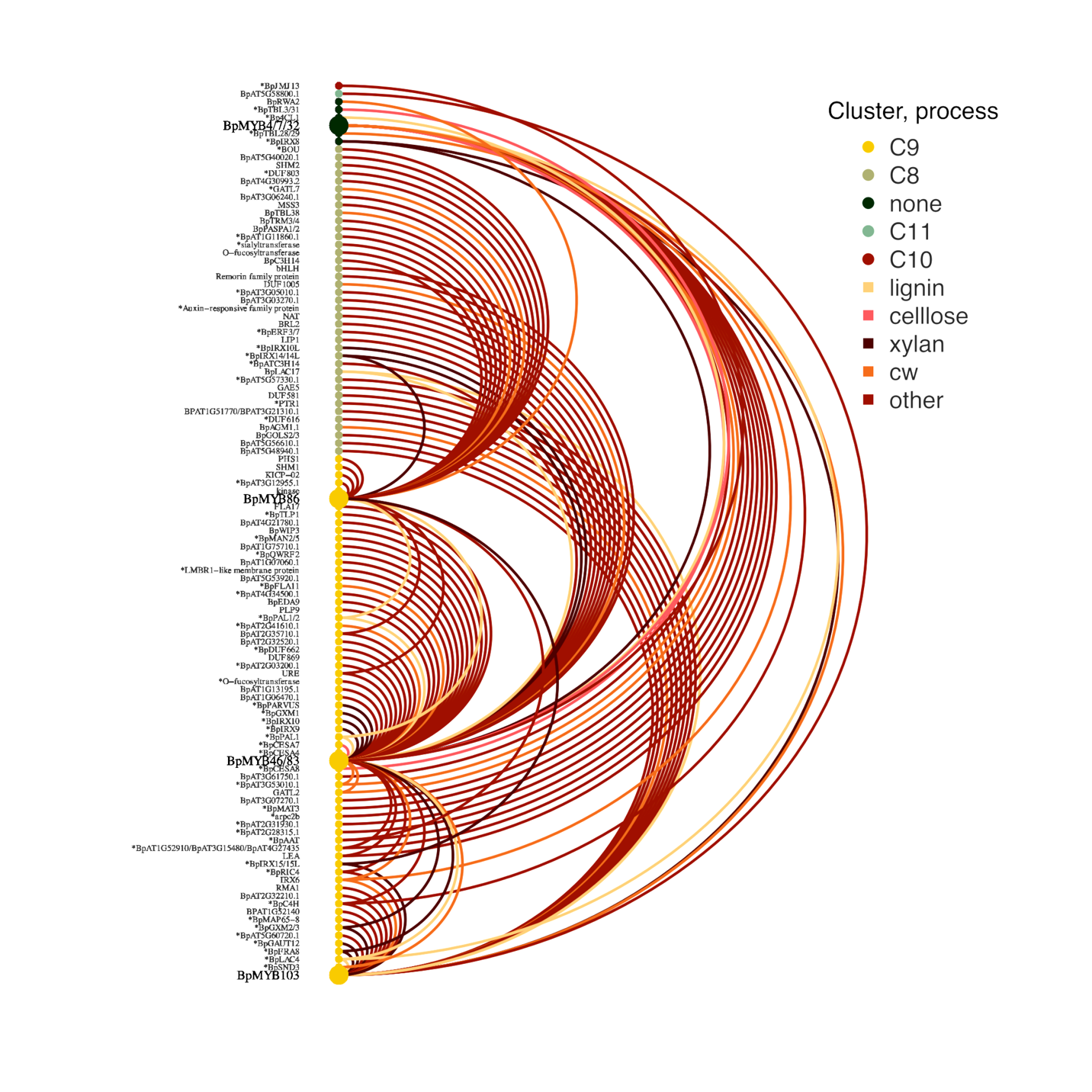


**Fig. S7** Gene tree of the second layer MYB transcription factors orthogroup.

Gene tree of the second layer MYB transcription factors orthogroup. Protein sequences were aligned with MAFFT and gene tree was estimated with FastTree.


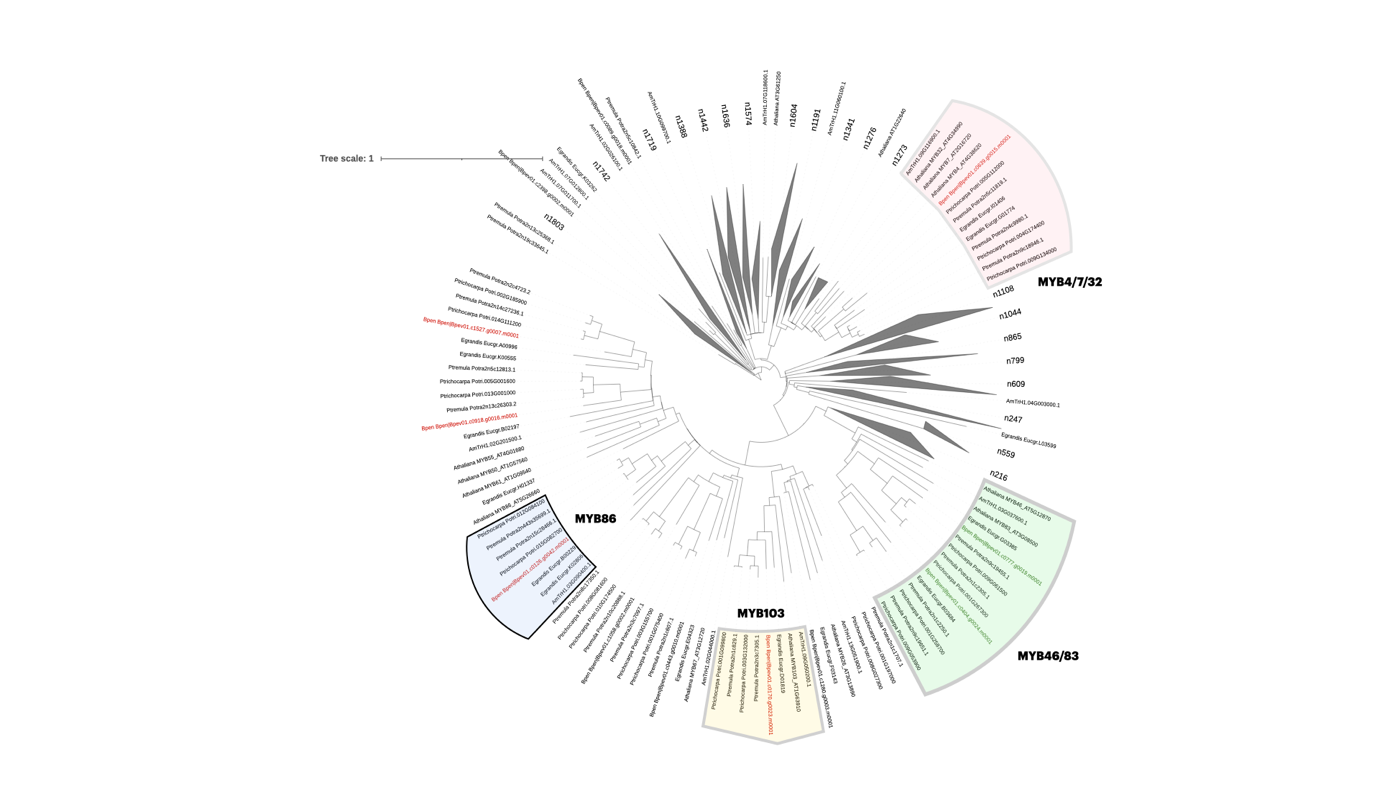


**Fig. S8** Gene tree of the xylan backbone genes IRX10, IRX10L.

Gene tree of the xylan backbone genes IRX10, IRX10L. Orthogroups and rooted pyhlogenetic trees were obtained with Orthofinder. Orthofinder was run with default settings using MAFFT for multiple sequence alignment and FastTree for gene tree inference.

**Fig. S9** Phylogenetic tree of the xylan backbone gene IRX9 orthogroup.

Phylogenetic tree of the xylan backbone gene IRX9 orthogroup. Protein sequences were aligned with MAFFT, and gene tree was estimated with FastTree.

**Fig. S10** Phylogenetic tree of the xylan backbone gene IRX9L orthogroup.

Phylogenetic tree of the xylan backbone gene IRX9L orthogroup. Protein sequences were aligned with MAFFT, and gene tree was estimated with FastTree.

**Fig. S11** Expression of the *BpIRX9L* and a potential paralog from the same orthogroup.

**Fig. S12** Phylogenetic tree of the xylan backbone genes *IRX14, IRX14L* orthogroup.

Phylogenetic tree of the xylan backbone genes *IRX14, IRX14L* orthogroup. Protein sequences were aligned with MAFFT, and gene tree was estimated with FastTree.

**Fig. S13** Expression of the *BpIRX14/14L* and a potential paralog from the same orthogroup.

**Fig. S14** Gene tree of the xylan glucuronylation genes, GXM orthogroup.

Gene tree of the xylan glucuronylation genes, GXM orthogroup. Protein sequences were aligned with MAFFT, and gene tree was estimated with FastTree.

**Fig. S15** Phylogenetic tree of the xylan acetylation genes RWA orthogroup.

Phylogenetic tree of the xylan acetylation genes RWA orthogroup. Protein sequences were aligned with MAFFT, and gene tree was estimated with FastTree.

**Fig. S16** Xylan biosynthesis in *B.* pendula and predicted regulators*.*

Predicted regulators of secondary cell wall xylan biosynthesis in *B. pendula*. Backbone biosynthesis genes *IRX9/10/14/14L*, reducing end biosynthesis genes: *PARVUS*, *IRX8*, *IRX7*; transport of acetyl-CoA: *RWA1/3/4*; Acetyl transferease TBL genes; glucuronylation *GUX1/2* genes; methyl transferase genes *GXM1/2/3*; deposition of xylan *IRX15/15L*.

**Fig. S17** GO BP enrichment of the orthogroups in the SCW cluster 4, based on clustering of gene expression datasets from multiple species, with CLUST.

**GO BP enrichment of the orthogroups in SCW cluster 4, based on clustering of gene expression datasets from multiple species using CLUST.** There are 54 orthogroups (OGs) containing genes from all species, and 39, 6, and 30 OGs containing genes exclusively from E. grandis, B. pendula, and P. trichocarpa, respectively. In all orthogroups, E. grandis and P. trichocarpa have a higher number of genes than B. pendula. Significantly enriched GO categories are listed for each group (Bonferroni-adjusted p < 0.05). The 30 OGs specific to P. trichocarpa did not have significantly enriched terms.

**Fig. S18** *Populus trichocarpa*, expression profiles of the genes for each cluster.

*Populus trichocarpa*, expression profiles of the genes for each cluster, as generated by CLUST. The expression of the replicates is averaged per fraction, and normalized with CLUST using quantile normalization, z-score and log2 transformation. The cluster size is in brackets.

**Fig. S19** Expression (TPM) profile of *BpKNAT7* and *BpKNAT3.*

Expression (TPM) profile of *BpKNAT7* and *BpKNAT3*. The dashed vertical lines point to the fractions where the secondary cell wall is deposited.

**Fig. S20** GO biological processes and molecular function enrichment of the genes predicted to be co-regulated by *BpKNAT7* and *BpKNAT3.*

GO biological processes and molecular function enrichment of the genes predicted to be co-regulated by *BpKNAT7* and *BpKNAT3*. Concurrent regulation refers to the case when *BpKNAT7 and BpKNAT3* have the opposite sign in the regression coefficient and together when they have the same sign. The circle size corresponds to the number of genes annotated with the given term.

**Fig. S21** Expression (TPM) of the PCW biosynthesis genes in the birch orthologs.

Expression (TPM) of the PCW biosynthesis genes in the birch orthologs**. (a)** Backbone biosynthesis *BpIRX9L, BpIRX10L***; (b)** Glucuronylation *BpGUX3*. The dashed vertical lines mark the fractions where the secondary cell wall is deposited.

**Fig. S22** Monomeric sugar content (% dry weight) across the stem fractions.

Monomeric sugar content (% dry weight) across the stem fractions. Ara – arabinose, GlcA – glucuronic acid, Man – manose, Rha – rhamnose, MeGlcA – methyl glunucronic acid, Gal – galactose, Xyl – xylose, GalA – galacturonic acid, Glc – glucose.

**Fig. S23** PCA of the monomeric sugar content.

PCA of the monomeric sugar content, with PC1 on x-axis and PC2 on y-axis. Cos2 indicates the importance of the principal component for the given individual, with high cos2 values indicating good representation of the variable on the component.

**Fig. S24** Pearson correlation of the clusters mean expression and the sugar content (% dry weight).

Pearson correlation of the clusters mean expression and the sugar content (% dry weight). Clusters as obtained with CLUST. Ara – arabinose, GlcA – glucuronic acid, Man – manose, Rha – rhamnose, MeGlcA – methyl glucuronic acid, Gal – galactose, Xyl – xylose, GalA – galacturonic acid, Glc – glucose. Clusters as obtained by CLUST.

**Fig. S25** PCA of transformed sugar profiles and Pearson correlation.

**(a)**. PCA of the transformed sugar profiles. The sugar profiles were transformed so that the difference of the concentrations between consecutive pairs of tissues was calculated, using the vascular cambium as the point of reference. **(b)**. Pearson correlation of the cluster mean expression and transformed the monomeric sugar concentrations.

|  |  |
| --- | --- |

**Fig. S26** Expression (TPM) of the orthologs of the Arabidopsis TBL gene family, involved in acetylation of cell wall.

Expression (TPM) of the orthologs of the Arabidopsis TBL gene family, involved in acetylation of cell wall.

**Fig. S27** Xylan oligosaccharide mass profiling analysis of developmental tissues in birch wood.

Xylan oligosaccharide mass profiling analysis of developmental tissues in birch wood. The alcohol insoluble residues (AIR) of tissue-specific wood fractions were hydrolysed by *Aa*GH10 endoxylanase and analyzed with AP-MALDI-ITMS. **(a)** Mass spectra for acidic pento-oligosaccharides liberated from young phloem, vascular cambium and young xylem. The relative abundance of acidic POS from all wood fractions were subjected to PCA analysis. **(b)** PCA plot showing separation of F2 to F8. **(c)** Loading of PC 1 (75% variance) showing mass peaks that separate young phloem from xylem tissues. **(d)** Loading plot PC 2 (11% variance) showing mass peaks that separate young xylem from matured and last year xylem. P_X,_ pentosyl residues; 1…6, number of acetyls; *, main peak(s); #, internal standard.

| **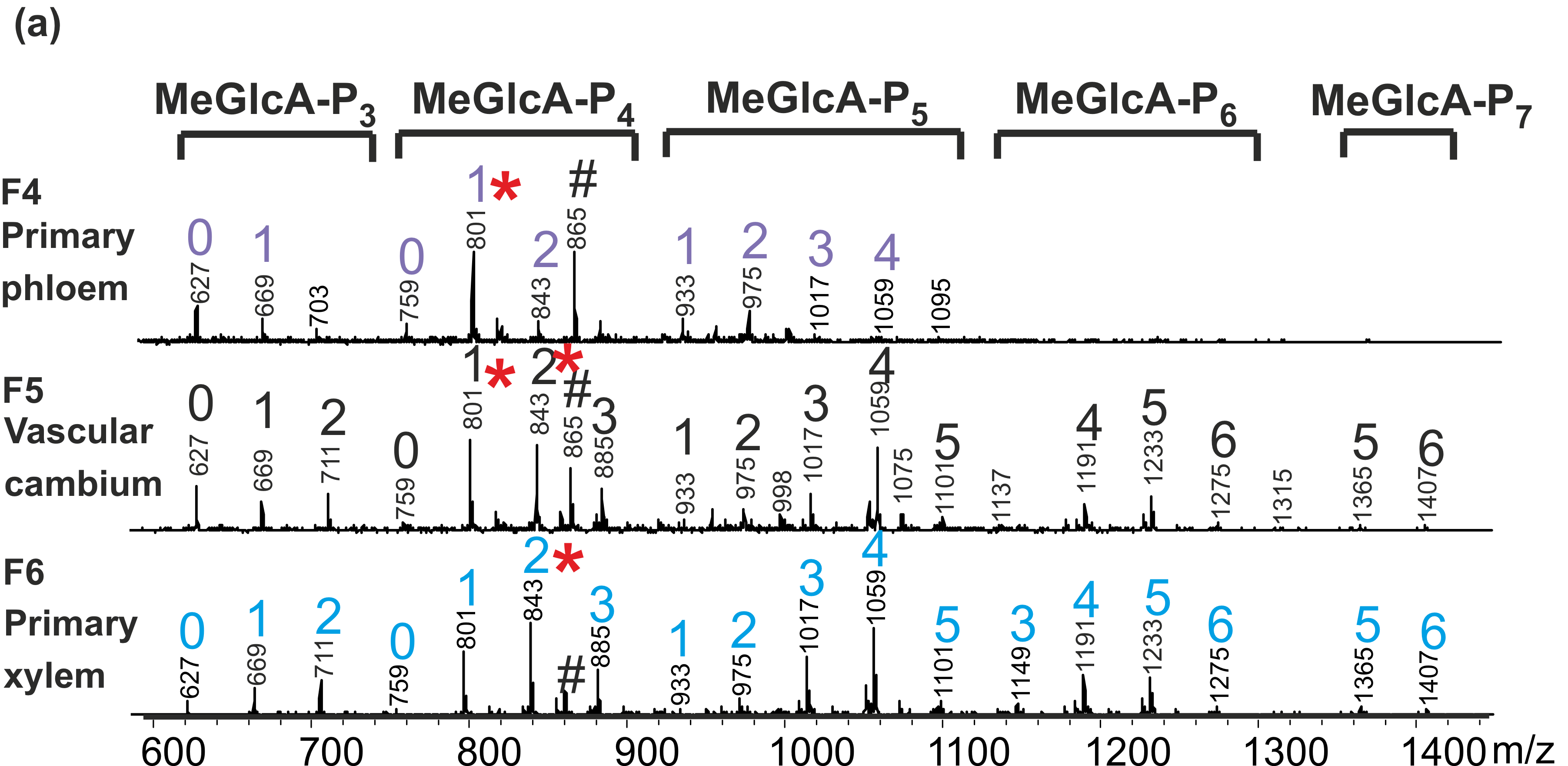** | **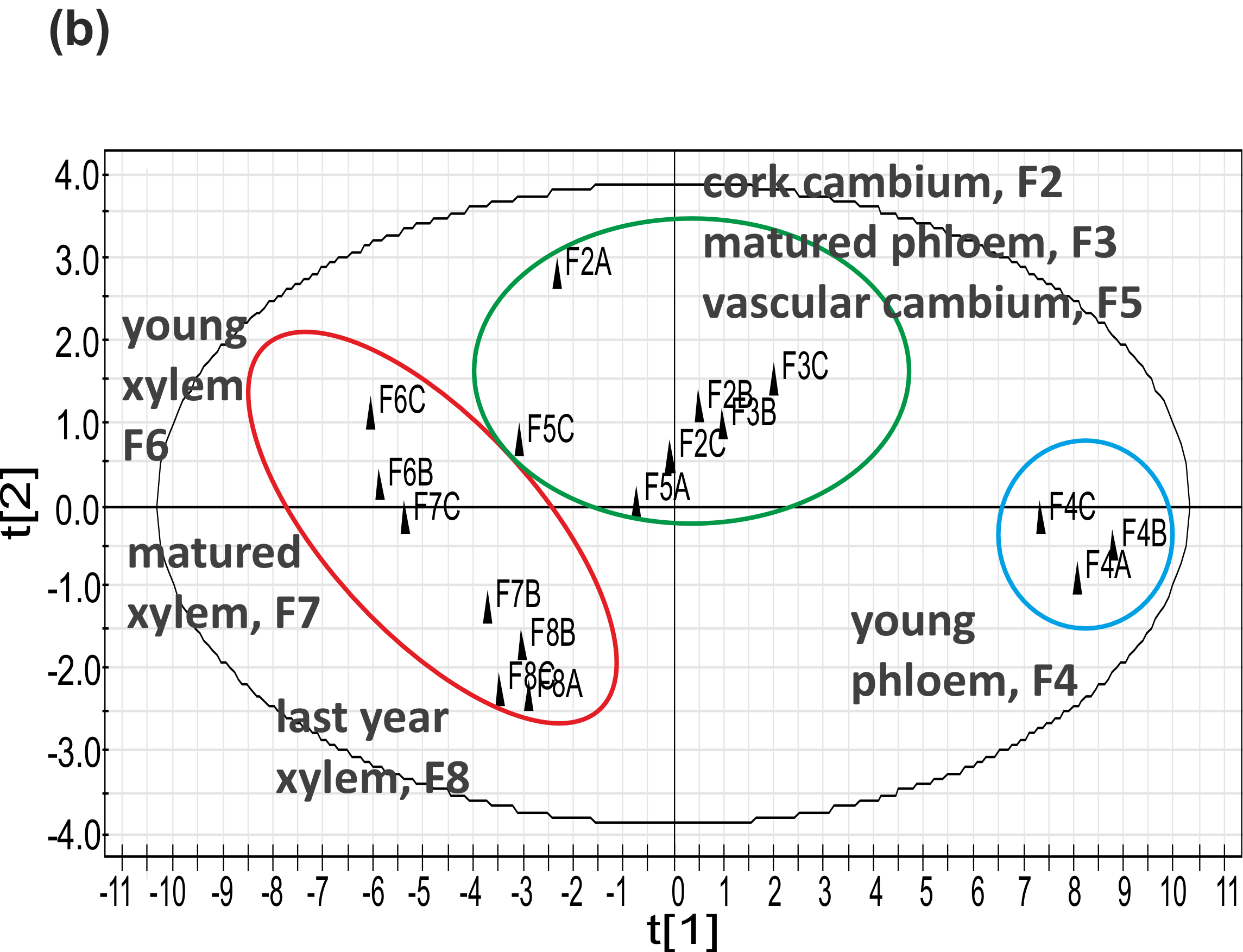** |
| --- | --- |
| **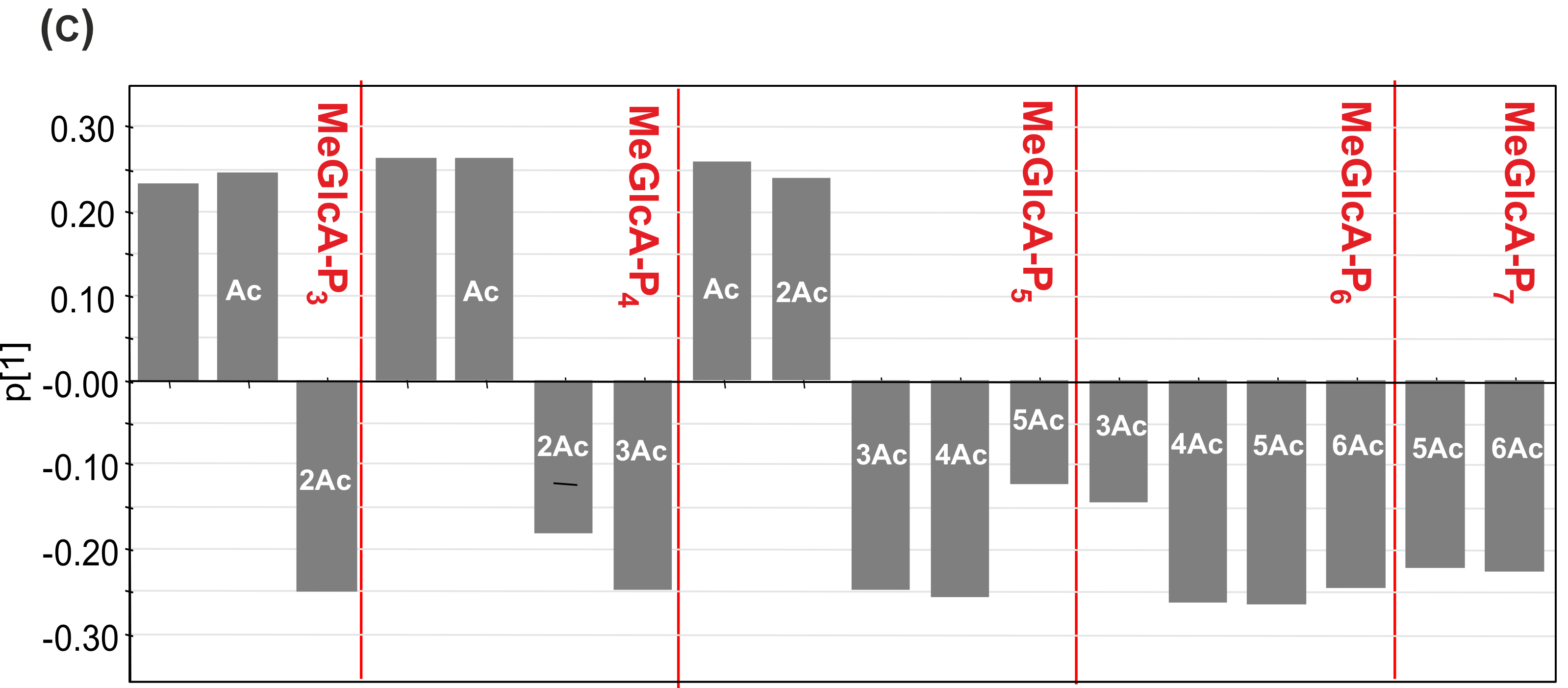** | **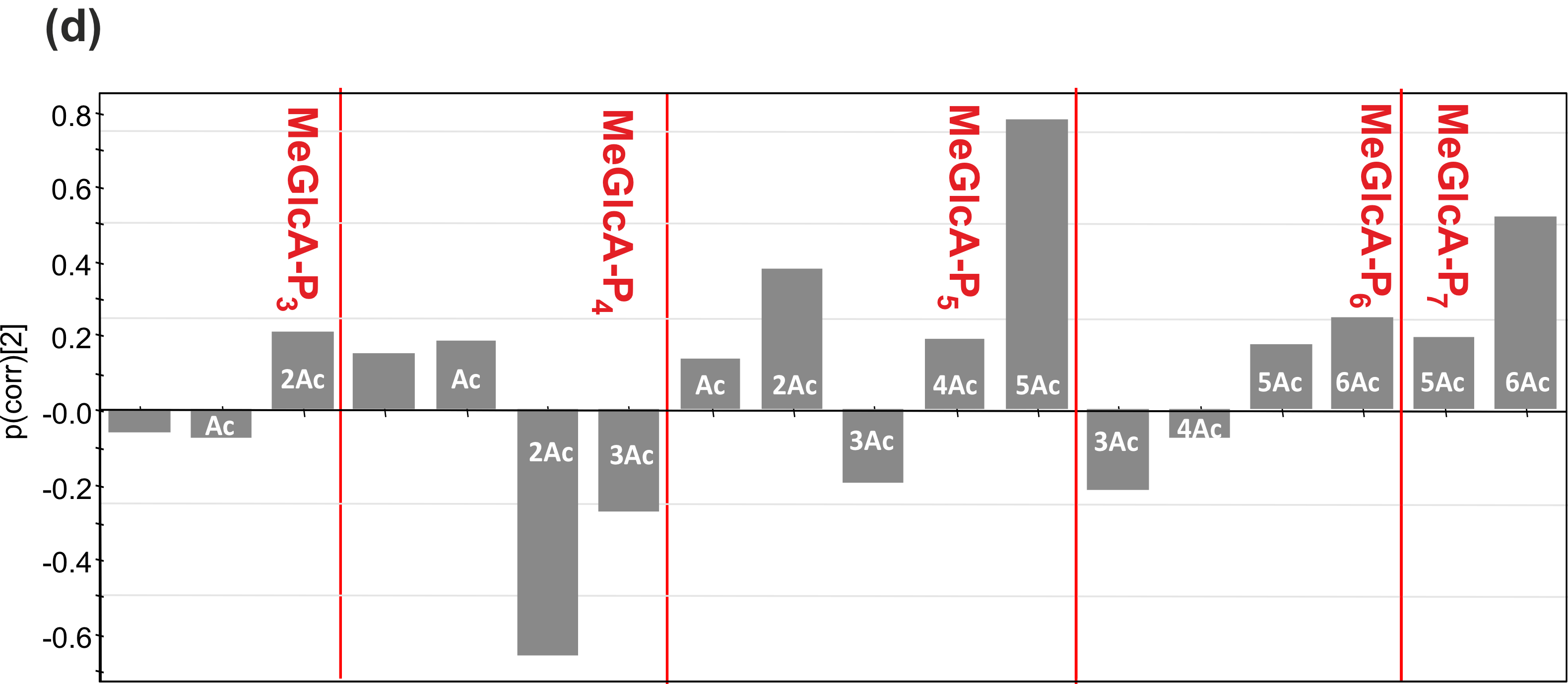** |

**Fig. S28** Structural study of the main peak (MeGlcA-P_4_Ac) present in the acidic fraction of AaGH10 endoxylanase hydrolysate from young phloem.

Structural study of the main peak (MeGlcA-P_4_Ac) present in the acidic fraction of AaGH10 endoxylanase hydrolysate from young phloem**. (a)** MS^n^ analysis of MeGlcA-P_4_Ac (m/z 777) in negative ion ESI-ITMS. **(b)** The possible isomeric structures deduced from the MS^n^ analysis.

**
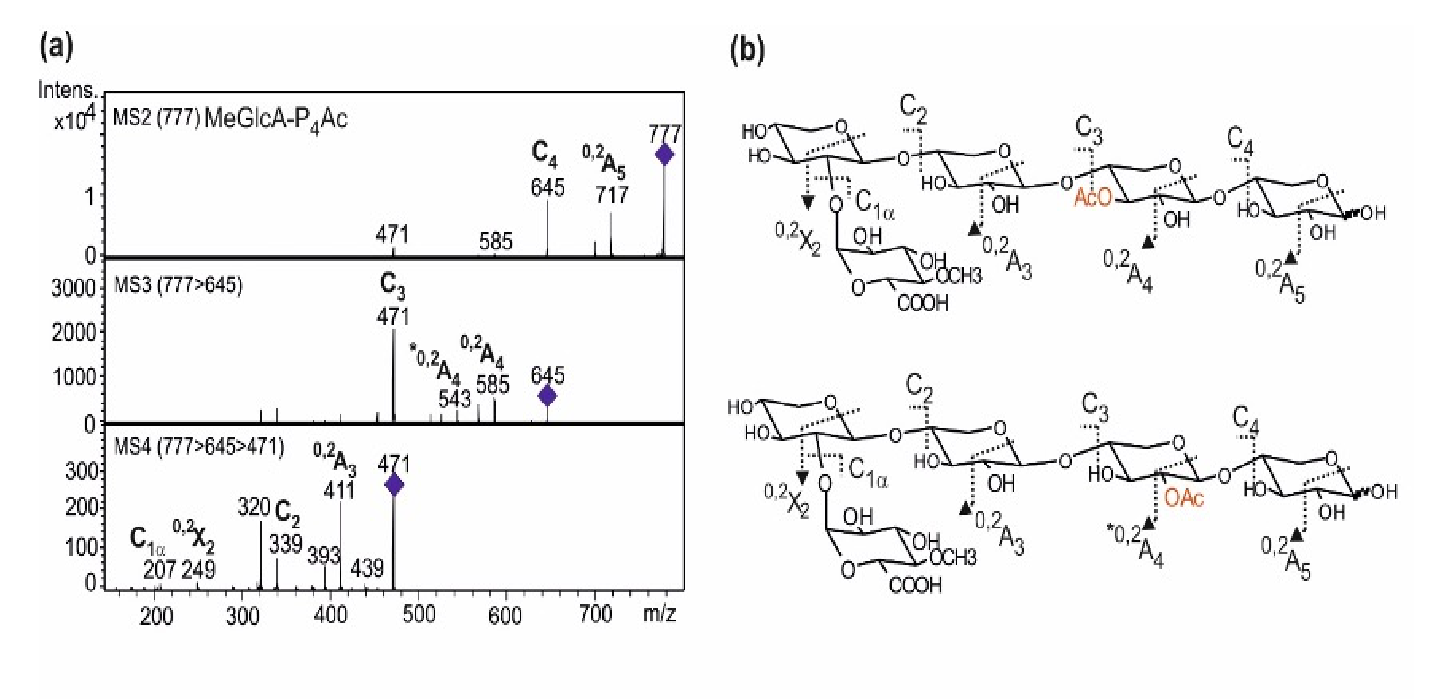
**

**Fig. S29** Structural study of the minor peak MeGlcA-P_5_Ac present in the acidic fraction of *Aa*GH10 endoxylanase hydrolysate from young phloem.

Structural study of the minor peak MeGlcA-P_5_Ac present in the acidic fraction of *Aa*GH10 endoxylanase hydrolysate from young phloem. **(a)** MS^n^ spectra of MeGlcA-P_5_Ac (*m/z* 909) in negative ion ESI-ITMS. **(b)** The possible structures deduced from the MS^n^ analysis.

**
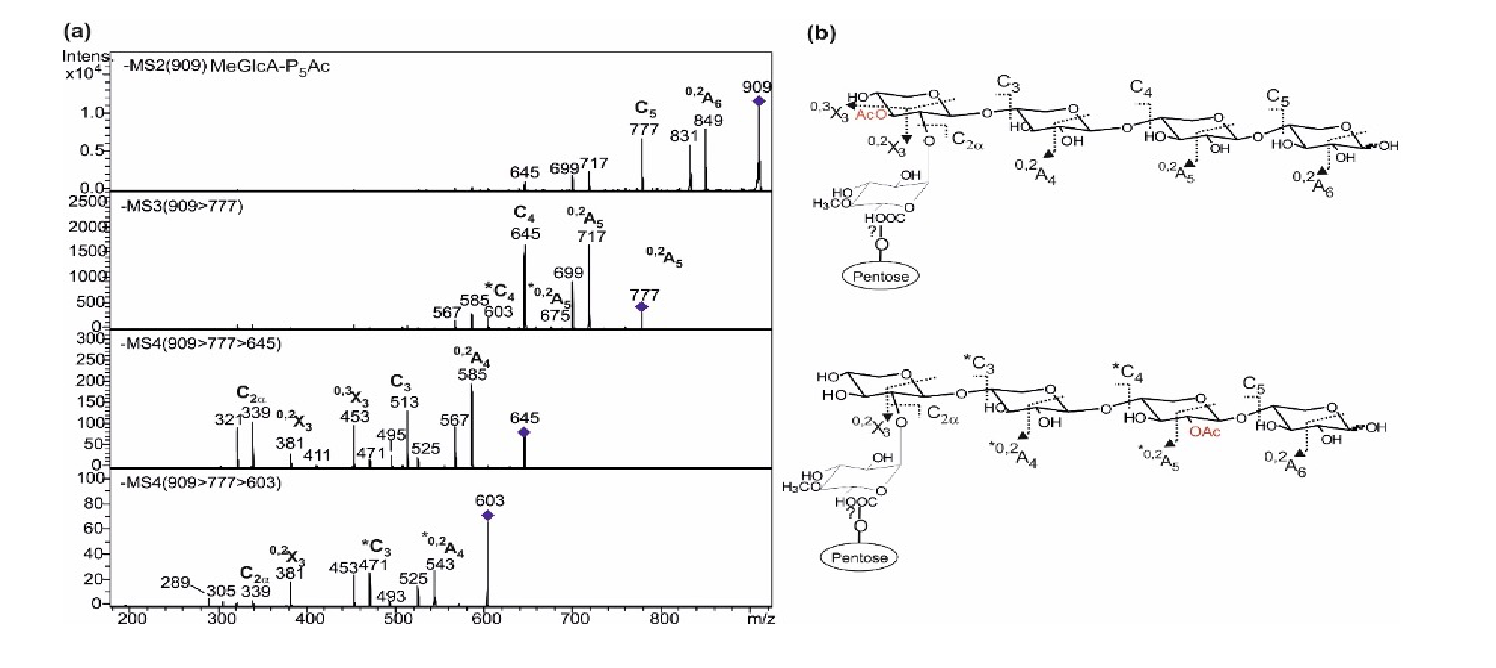
**

**Fig. S30** MeGlcA substitution pattern of birch young xylem and phloem xylan.

MeGlcA substitution pattern of birch young xylem and phloem xylan. Alkali pre-treated AIR was hydrolysed with glucuronoxylanase *Bo*GH30, derivatised with ANTS, and the resulting oligosaccharides were separated by polyacrylamide gel electrophoresis (PACE). X­_2-6­_ standard xylooligosaccharides. UX_3-16­ ­_singly [Me]GlcA-substituted GH30 product xylooligosaccharides described for xylan from *Arabidopsis thaliana* (At). * Oligosaccharide that comigrates with UX_6_. ** Oligosaccharide AU^Me^X_6_ with similar migration to the previously described PUX_6_ structure. *** Unidentified oligosaccharide product.

**
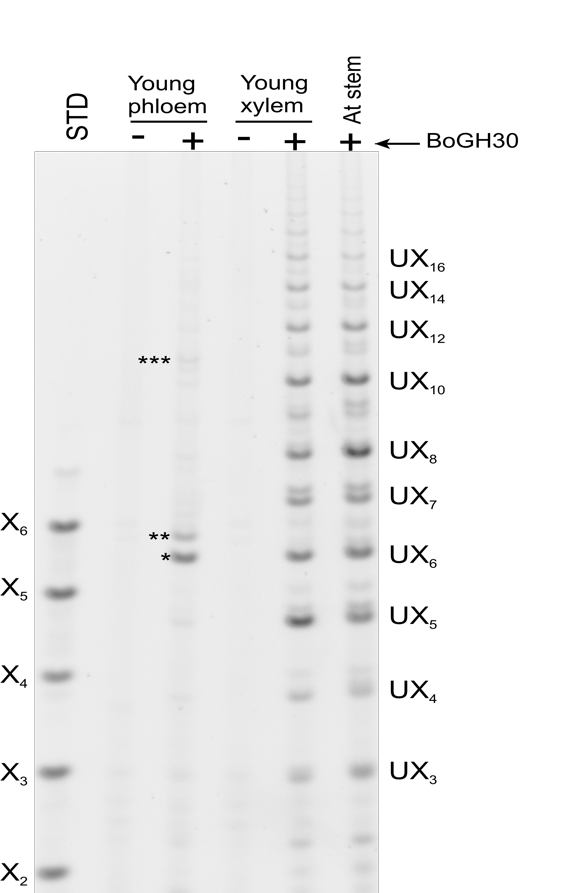
**

**Fig. S31** Comparison of qHSQC spectra of TrGH11 hydrolyzed O-acetylglucuronoxylans isolated from young xylem (red) and young phloem (black).

Comparison of qHSQC spectra of TrGH11 hydrolyzed O-acetylglucuronoxylans isolated from young xylem (red) and young phloem (black). X2, [2-O-Ac]-β-D-Xyl*p*; X3, [3-O-Ac]-β-D-Xyl*p*; X23, [2,3-O-Ac]-β-D-Xyl*p*; X3G2, [α-D-(Me)Glc*p*A(1→2)][3-O-Ac]-α-D-Xyl*p*; XG2, [α-D-(Me)Glc*p*A(1→2)]-β-D-Xyl*p*.

**
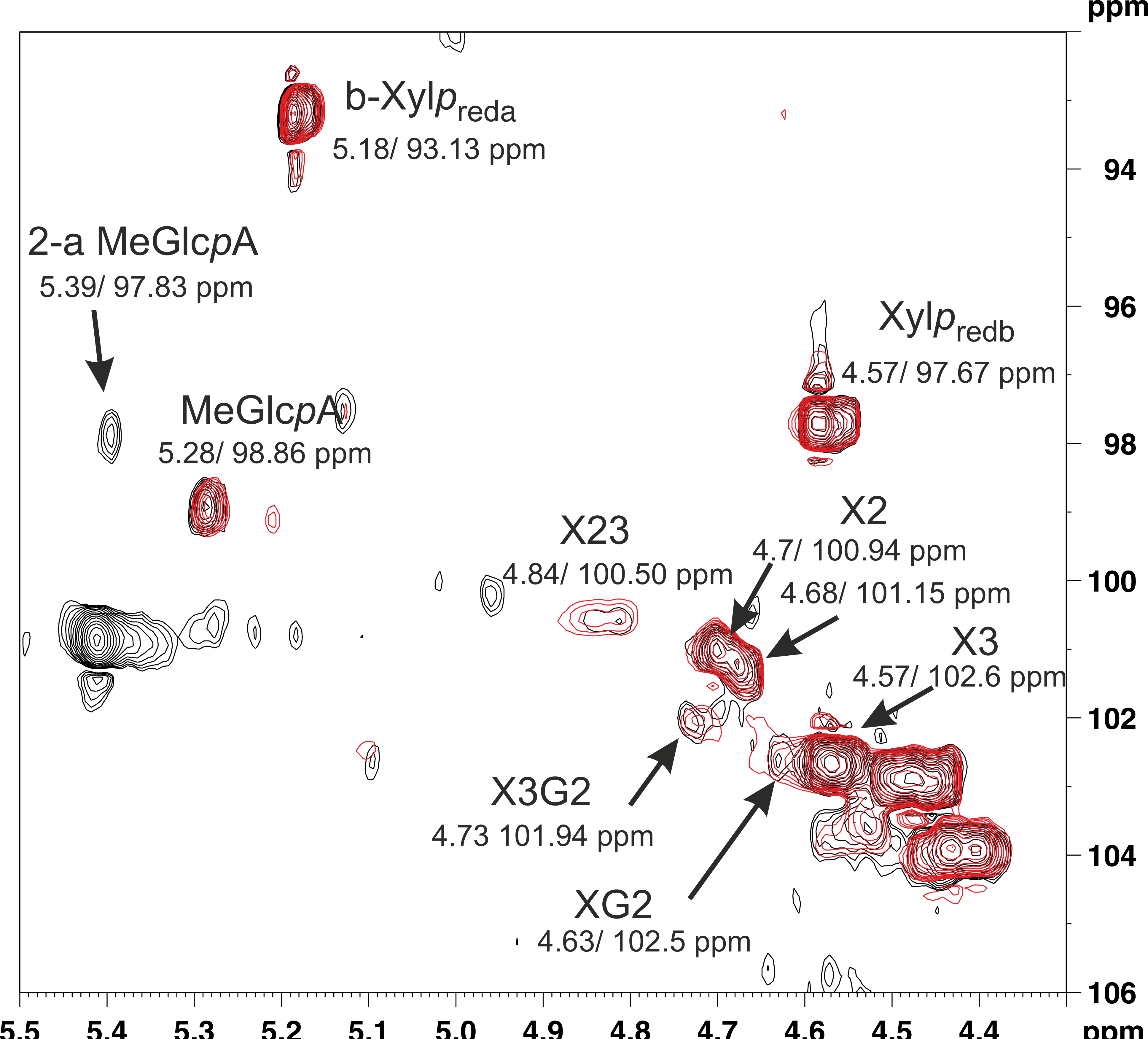
**

**Fig. S32** qHSQC spectrum obtained from the TrGH11 hydrolyzed deacetylated glucuronoxylans isolated from young phloem.

qHSQC spectrum obtained from the TrGH11 hydrolyzed deacetylated glucuronoxylans isolated from young phloem. The H1/C1 signals for β-Xyl*p*, α-MeGlc*p*A and α-Ara*p* were assigned according to (Peña et al., 2007, Pena et al., 2016)

**
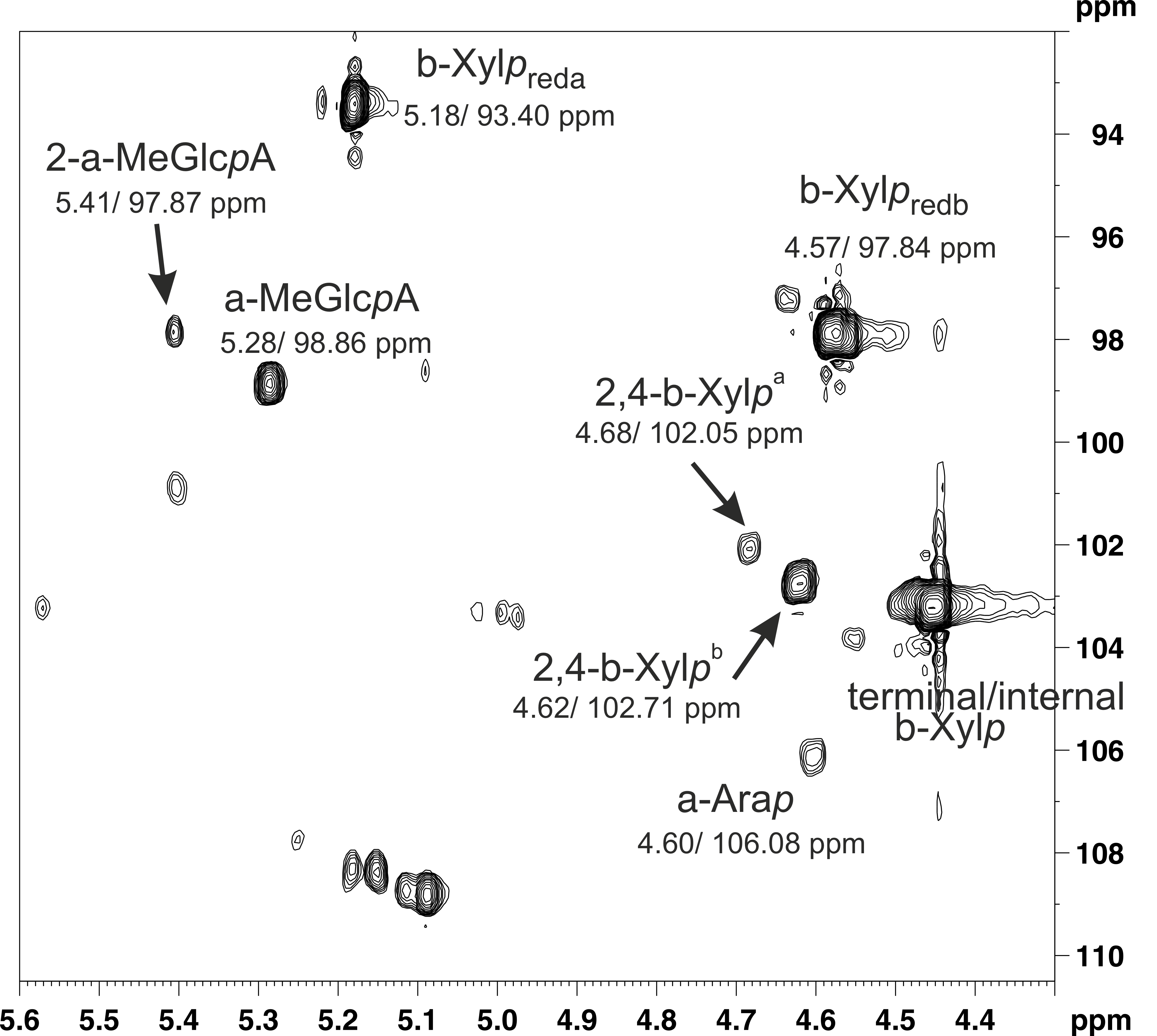
**

**Fig. S33** Nuclear Overhauser Effect Spectroscopy (NOESY) spectrum obtained from deacetylated glucuronoxylans isolated from young phloem.

Nuclear Overhauser Effect Spectroscopy (NOESY) spectrum obtained from deacetylated glucuronoxylans isolated from young phloem. The inter-glycosidic linkages α-Ara*p*-(1→2)-α-MeGlc*p*A-(1→2)-β-Xyl*p*-(1→ were established by detecting H1/H2’ cross peaks at 5.41/3.54 and 4.59/3.74.


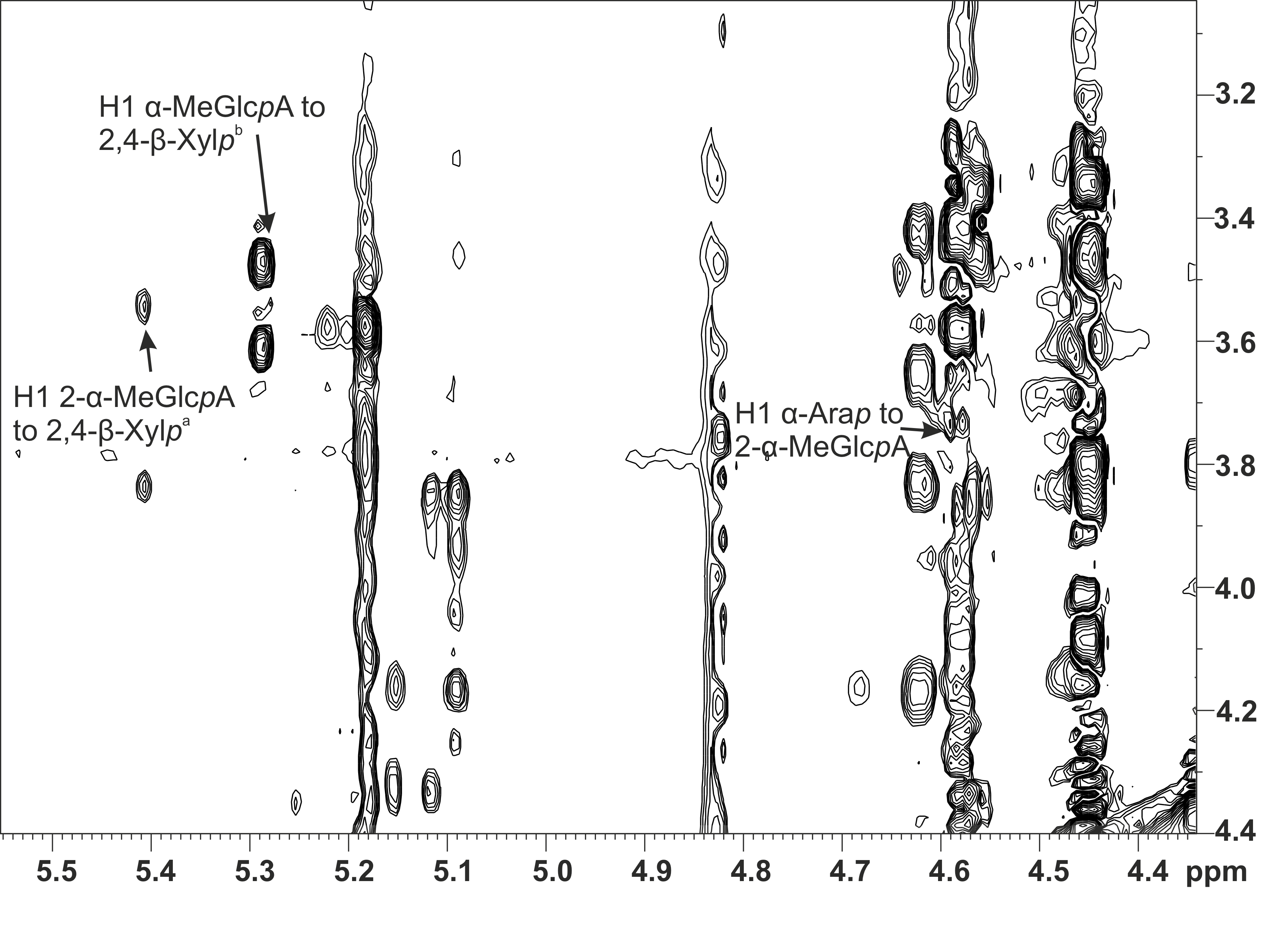


**Fig. S34** Gene tree of XAPT-like sequences.

Gene tree of XAPT-like sequences retrieved with HMMER and TBLASTN, using AtXAPT1 as query, and AtMUR3 for rooting. Protein sequences were aligned with MAFFT, and gene tree was estimated with RaxML.

**Fig. S35** Expression of the genes that correlate (absolute correlation > 0.7) with the P4Ac2 xylem MS peak.

**Fig. S36** Expression of the orthologs of xylan SCW biosynthesis genes in *B.pendula*.

Expression of the orthologs of xylan SCW biosynthesis genes in *B.pendula*. **(a)** glucuronoxylan backbone – *BpIRX9*, *BpIRX10*, *BpIRX14/14L*, (b) (Methyl) Glucuronic Acid – *BpGXM1*, *BpGXM2/3*, *BpGUX1*, *BpGUX2*, **(c)** Acetyl-transferases –*BpTBL28-35*, **(d)** reducing end – *BpFRA8/FH8*, *BpIRX8*, BpPARVUS and transport of acetyl-CoA *BpRWA1/3/4*.

**Fig. S37** Expression profile of the genes that correlate with the phloem MS peaks.

Expression profile of the genes that correlate (absolute correlation > 0.7) with the **(a)** P4Ac1 phloem MS peak and **(b)** with the P5Ac1 phloem peak.

**Notes S1** **Weighted Gene Co-expression Network Analysis** (**WGCNA) clustering**

Given the large number of unclustered genes with we further used WGCNA co-expression clustering method to extend the CLUST result to identify all genes that are functionally related. This resulted in 75 clusters with diverse set of enriched profiles, with cluster assignments for all genes. The majority of the genes were assigned to clusters with less than 1K genes, with few clusters having sizes between 1-8K. We performed GO enrichment analysis for the WGCNA clusters (Table S2). To combine the two clustering results, we further used Fisher’s exact test to identify significant overlaps between the two clustering results. Out of the 19 clusters, 16 had at least 70% of the genes clustered in WGCNA clusters with significant association to the original CLUST cluster (one-sided Fisher’s exact test; Table S3). The smallest two CLUST clusters were split into 5 WGCNA clusters, while the largest two were split into 12 and 13 clusters, respectively. In the main work, we describe the results based on CLUST in more detail without specifically referring to the method when we talk about clusters. Regarding the WGCNA results, we only focus on the clusters with genes related to cell wall biosynthesis, and we always refer to the method when doing so.

**Notes S2** **Xylan biosynthesis in *B. pendula* and regulation of cell wall biosynthesis**

Xylans, the manor hemicellulose in hardwood, are formed by (1→4)-linked-β-xylopyranosyl backbones that carry substituents such as (4-*O*-methyl) glucupyranosyluronic acid (MeGlc*p*A) and acetyl residue. Here, we looked at the biosynthesis genes in birch, their expression and phylogenetic trees. Based on computational clustering with orthofinder, the xylan backbone synthesis gene *IRX10* had two orthologs in *P. trichocarpa*, one ortholog in *P. tremula* (Potra2n1c546.1), and one in birch (**Figure S8**). The *IRX9* had one ortholog in birch, two in poplar and two in aspen (**Figure S9**). *IRX9L* had one predicted ortholog in the three species, birch, poplar, and aspen. The orthogroup with *IRX9L* contained another birch gene, for which the *Arabidopsis* copy seems to be lost (**Figure S10**). The presence of an ortholog of this gene in poplar, which has been identified as a second homolog of the *AtIRX9L* gene (Sundell et al., 2017), might indicate two copies of the *IRX9L* gene in woody plants. The expression of *BpIRX9L* peaks in F4, with high expression in F3 and F5, while the expression of the second gene peaks in F5. (**Figure S11**). The *IRX14* and the homolog *IRX14L* in Arabidopsis had one ortholog in birch, two in *P.trichocarpa* and three in *P. tremula* (**Figure S12**). This orthogroup also included a second birch gene branching off from the basal *Amborella* gene, with expression peaking in F3, while the expression of *BpIRX14/14L* peaked in F3 and F6 (**Figure S13**). Based on the amino acid sequence the second gene was identified as a member of the glycosyl transferase family 43, same as the *IRX14* and *IRX14L* genes. According to the Orthofinder results, *IRX10* and *IRX10-L* were in the same orthogroup (**Figure S8**), whereas *IRX9* and *IRX9-L* were in different. In terms of gene expression, *IRX9* and *IRX10* were in cluster 9 (Figure 2B) based on the CLUST method, *IRX10L* and *IRX14/14L* in cluster 8, whereas the *IRX9L* was not clustered. According to WGCNA, the secondary cell wall *IRX10, IRX9* and *IRX14/14L* were in the same cluster – cluster 6, with the primary cell wall *IRX9L* and *IRX10L* in clusters 12 and 17 respectively (Figure 2B). All the birch backbone synthesis homologs, but the two IRX9L copies and one of the *IRX14/14L* genes, have a xylem specific SCW expression peak (**Figures S36**).

Reducing end-related genes *FRA8* (*IRX7*) and *F8H* (*IRX7L*) were assigned to the same orthogroup, with one common ortholog in *B. pendula*, while the *IRX8* and the *PARVUS* belong to two different orthogroups. *IRX8* (putative galacturonosyltransferase) was grouped with *GAUT13* and *GAUT14*, galacturonosyltransferases potentially involved in pectin synthesis of the pollen tube wall (Wang et al., 2013). *PARVUS* was assigned to the same orthogroup (OG3709) as *GATL2*, a second galacturonosyltransferase involved in pectin biosynthesis. All the reducing end synthesis genes were assigned to a secondary cell wall specific cluster 9 (Figure 2B), or cluster 6 with WGCNA. *GATL2* also belongs to cluster 9, with expression profile typical of SCW.

SCW genes decorating glucuronic acid on xylosyl residues, *BpGUX1* and *BpGUX2* were in two different orthogroups and in cluster 8 and 9, respectively. In the same orthogroup with *BpGUX1* was *BpGUX3* the ortholog of the PCW *GUX3* gene, clustered in cluster 19.

The three genes controlling the methylation of xylan, GXM*1*, *GXM2* and *GXM3*, are all expressed in secondary wall forming tissues. All these genes were grouped to the same orthogroup with the *GXM2* and *GXM3*, likely paralogs, with three *P. trichocarpa* genes and one *B. pendula* gene, respectively (**Figure S14**). Another *Arabidopsis* gene (AT1G71690) encoding glucuronoxylan 4-O methyltransferase-like protein is grouped in the same orthogroup, as an outgroup to the *GXM* genes, splitting after the *A. trichopoda* gene. The birch and poplar genes have very similar expressions, with peak in the developing xylem as well as peak in F3 in the birch genes. One of the poplar genes has a peak in the mature xylem. All 3 genes were clustered in cluster 9 (**Figure 2B**), whereas they belong to cluster 6 and cluster 12 with WGCNA. The three methyltransferases *GXM1*, *GXM2* and *GXM3*, catalyzing 4-O-methylation of GlcA side chains in xylan, have the unknown domain DUF579. Besides the GXM genes, in *Arabidopsis,* there are two DUF579 genes, namely *IRX15* and *IRX15L* involved in xylan deposition. However, their enzymatic activity is unknown (Brown et al., 2011). In birch there is one ortholog of both genes which belongs to cluster 9.

Transport of acetyl-COA genes, *RWA1-4*, are all clustered in the same gene orthogroup (**Figure S15**). The *RWA2*, involved in acetylation of the primary cell wall in Arabidopsis (Manabe et al., 2011), is in a branch of the gene tree with two birch homologs (tandemly duplicated) while the remaining RWA genes related to secondary cell wall are found in the sister clade with one ortholog in birch. There were two homologs in poplar and aspen corresponding to the *RWA2* and *RWA1-4*. With CLUST, the birch ortholog of *RWA1/RWA3/RWA4* was not clustered – even though its expression is peaking in SCW fractions, the pattern across all fractions is deviating from cluster 9 expression. One ortholog of the PCW *RWA2* was present in cluster 9, while the other one was filtered out due to low expression. With WGCNA, the *RWA1/3/4* and the expressed homolog of *RWA2* were placed in cluster 6 (**Figure 2B**).

*MYB58/MYB63* are known transcriptional regulators of lignin biosynthesis in Arabidopsis. Based on orthofinder, there is one birch gene ortholog of the *Arabidopsis* *MYB58*, *MYB63*. The gene is having a high peak in the phloem (F3), whereas no expression in the SCW xylem tissues. We checked the presence of the MYB58/63 specific binding motif and the genes for which the motif was present were enriched in cell wall-specific processes. More specifically, xylan, lignin, and cellulose biosynthesis, SCW biosynthesis, but also PCW pectin biosynthesis. This could suggest that BpMYB58/63 is a regulator of cell wall in phloem tissues, but not active in birch xylem. Some of the genes with the motif are annotated with stress response processes, however, those terms were not significantly enriched among the set of genes with present motifs. The xylan SCW biosynthesis genes and their predicted regulators based on the network analysis are summarized in **Figure S16**.

**Notes S3** **KNAT7 is a putative regulator of lignin biosynthesis**

Four members of the KNAT family, *AtKNAT3*, *AtKNAT4, AtKNAT5* and *AtKNAT7* were clustered in the same orthogroup, with *AtKNAT3* and *AtKNAT4* having a single representative in birch. The birch ortholog of *AtKNAT7* did not have the binding motifs of SW NACs in its promoter region 3kb upstream of TSS, nor the MYB-specific binding motifs, suggesting indirect regulation. However, the SW NAC binding motif occurred at least three times in the promoter region of the ortholog of the *AtKNAT3* and *AtKNAT4*, and the occurrence of the MYB-specific binding motifs was detected.

Chromatin immunoprecipitation with quantitative PCR on promoter sequences of the *IRX7* and *IRX14* downstream from KNAT has suggested the [KBS, TGACAG(G/C)T] sequence as the possible binding site of *KNAT7* (Wang et al., 2020). However, no presence of the KBS motif in the promoters of the putative birch orthologs of *KNAT7* targets in Arabidopsis was found. It is possible that the binding specificity of *KNAT7* could be tissue-specific and the binding motif different in birch. Given that recent articles suggest a synergistic *KNAT3*/*KNAT7* regulation of the monolignol biosynthesis (Qin et al., 2020), we applied linear regression to identify potential targets of the KNOX II TFs by predicting their gene expression using both *KNAT7* and *KNAT3* as covariates. To test for regulatory interactions, we constructed models that either use only *KNAT7* or *KNAT3* as a covariate, an additive model including both genes and then a full linear model also including their interactions. The best linear model was identified with likelihood ratio tests. The precondition here is that the models are nested; the more complex model always contains the reduced model. A simpler model was preferred in the case where the likelihood ratio test yielded a p-value above 0.05 when compared to the more complex model, and the adjusted R-Square of the simpler model was above 0.7.

Altogether 51 genes were identified where expression was significantly explained by the additive model of knat7 and knat3 (+), a positive knat7 coefficient was fitted for 18 of those genes. The expression of the ortholog of *C4H* was explained by a positive combination of both regulators, whereas the ortholog of *F5H* fit displayed a positive coefficient for *KNAT7* and a negative for *KNAT3*. When a negative coefficient was fitted for *KNAT7*, the *KNAT3* coefficient was always positive and the target genes were enriched for processes related to stress response and acclimation: abiotic stimulus, heat acclimation, redox processes, stress response and regulation of cellular macromolecule biosynthetic process, response to environmental stimulus, primary metabolic process, cellular homeostasis, (negative) regulation of gene expression and response to carbohydrate. In the interactions where the interaction term knat7*knat3 was included, the downstream genes were enriched for phenylpropanoid and lignin biosynthesis (**Figure S20**, Table S19, S20). The same processes were enriched among the genes explained only by the expression of knat7. Among the genes explained by the reduced model of knat7, 21 in total, are multiple lignin genes, *BpMYB85*, few kinases and multiple genes with no validated function (Table S22). There are no genes from the xylan biosynthesis pathway identified with this approach that would be regulated solely by knat7. In addition, the predicted interaction can be seen as other type of interaction but the regulatory relationship that we assume, like synergistic regulation between KNAT genes and the target gene.

**Notes S4** **Xylan concentration, monomeric sugar composition**

Primary cell walls, dry weight, consist of 15–40% cellulose, 30–50% pectic polysaccharides, and 20–30% xyloglucans and lesser amounts of arabinoxylans and structural proteins (Cosgrove & Jarvis, 2012). Cellulose is major component of SCW as well – about 45- 50%, with 20-30% glucuronoxylan, 20% lignin and up to 5% glucomannan. The concentration of non-cellulosic sugars, namely arabinose (Ara), glucuronic acid (GlcA), manose (Man), rhamnose (Rha), methyl glucuronic acid (MeGlcA), galactose (Gal), xylose (Xyl), galacturonic acid (GalA) and glucose (Glc), in our samples was quantified by acid methanolysis and gas chromatography, and their content (% dry weight) was reported (**Figure S22**). The concentrations of all sugars except Xyl, MeGlcA and Man peaked in F4 and F5 - tissues abundant in PCW. In principal component analysis (PCA), reflecting their differential sugar compositions, the first principal component of the sugar content separates the PCW, namely F4 and F5, from the remaining fractions (**Figure S23**), explaining approx. 58% of the variance. The second PC separates F1 and F8 from other fractions and from each other, with F4 and F5 being close but separated and F2-F3, F6-F7 clustered together. We correlated the concentration data between F2-F8 with the average expression of each cluster. This resulted in clusters 15, 16 and 17 (**Figure 2B**) having high positive correlation with the monomeric sugars GalA, Glc and Ara, associated with PCW non-cellulosic components (**Figure S24**); cluster 16 was found to be enriched for cell wall biogenesis and biosynthesis of the components of the PCW in GO enrichment analyses. More specifically, genes like the cellulose PCW synthases *BpCESA1*, *BpCESA3* and *BpCESA10*, the xyloglucan backbone synthesis genes *BpCSLC5/8* and xyloglucan xylosyltranserases *BpXXT1* and *BpXXT2*, as well as the mannan acetylation TBLS, *BpTBL23/24*, were in cluster 16 – all of which are related to PCW. In addition, cluster 16 was enriched for syntenic duplicates and while there were no significant GO terms among those genes, interestingly they were involved in processes related to cell wall pectin biosynthesis - more specifically pectin lyases and cell wall modification involved in multidimensional cell growth, involving expansins. Both, lyases and expansins have been associated with change in cell wall architecture and growth, as well as modification (Li et al., 2003; Rose et al., 2004).

According to GO enrichments, clusters 15 and 17 (Figure 2B) were related to cell cycle and phloem development, respectively, and not directly to cell wall. Their high correlation with the PCW sugars is due to the expression pattern of those clusters peaking in the same fractions that are associated with PCW.

Also, clusters 1 and 19 (Figure 2B) were correlated with some of the PCW sugars, even though the correlations were not as high as in the previous clusters. One of the four xylan primary cell wall genes was in cluster 19 – the glucuronylation gene GUX3, with expression peaking in F2. This may suggest that the expression of the xylan primary cell wall biosynthesis genes has an additional (or sometimes single) peak in fraction 2 (cork cambium), unlike the expression of the other PCW genes. Homologs of the genes involved in the biosynthesis of the PCW components, including cellulose and xyloglucan, were in cluster 16. Cluster 16 was also enriched for pectin biosynthesis, all of which suggests that even though the cluster is not specifically related to xylan it has a profile specific to all the other PCW polymers. However, given that one of the orthologs of the known *Arabidopsis* PCW genes, namely GUX3 is in cluster 19 we still observe some relation between the concentration of the PCW sugars and the PCW xylan biosynthesis. Both cellulose and xylan contribute to the primary and secondary cell wall, with cellulose PCW genes in cluster 16 while xylan PCW genes were placed in clusters 8 and 19. Cellulose content is high in both primary and secondary cell wall, and there are separate CESA genes for PCW and SCW cellulose synthesis, which is probably why the genes have tissue specific profiles and separate into PCW and SCW specific clusters, namely cluster 16 and cluster 9 (Figure 2B). In the case of xylan, the backbone synthesis genes of PCW and SCW are partially functionally redundant and show partially overlapping expression patterns in *Arabidopsis* tissues with the PCW genes showing more widespread expression pattern compared to the SCW *IRX9*, *IRX10* and *IRX14* (Mortimer et al., 2015; Wu et al., 2010).

The PCA first and second component did not separate SCW tissues from the others, possibly because the observed abundances are accumulative when the tissue develops whereas gene expression is localized to the tissue type. Hence, we calculated the change of sugar concentrations between the consecutive pairs of tissues, using the vascular cambium as the point of reference. After the transformation, the first component of the PCA separated F3 and F6, namely the old phloem and developing xylem from the other tissues. This may suggest that F3 and F6 are the tissues with SCW specific profiles whereas the F2 and F7 although containing SCW, have rather mixed profiles (**Figure S25**). Correlating the transformed sugar concentrations with the mean expression of the clusters identified clusters 7 and 8 with high correlations with the Xylose and MeGlcA, and with low or negative correlations with the sugars associated with PCW.

Cluster 8 (Figure 2B) was enriched for xylan biosynthesis, which is the major non-cellulosic component of the SCW. Multiple lignin biosynthesis genes are in cluster 7, with some of them being tandemly duplicated. Cluster 8 was enriched for tandem duplicate genes including the *BpOMT1* – a methyltransferase involved in lignin synthesis, *BpGAUT11*, and *BpGAUT15* involved in pectin biosynthesis. In addition, the ortholog of Arabidopsis *AXY9* – a gene involved in acetylation of xyloglucan and xylan, and xylan catabolic gene *BpBXL2* are in cluster 8.

Out of the acetyltransferase genes involved in cell wall acetylation, *TBL3* and *TBL31* are required for O-3 acetylation of xylan (Yuan et al., 2016) and *AXY4/TBL27* and *AXY4L/TBL22* are required for O-acetylation of xyloglucan (Gille et al., 2011). The expression of the ortholog of *TBL27* peaks in F4 implicating participation in PCW synthesis, while the ortholog of *TBL22* was removed due to low expression (**Figure S26**). In addition to these TBL members, *TBL29* (*ESKIMO1*) mediates xylan O-acetylation (Urbanowicz et al., 2014; Xiong et al., 2013). *TBL1/2/4/5/6* and *TBR* are clustered in the same orthogroup, with the *TBR* gene assigned as a putative ortholog of *TBL1*. *TBL37-43* have been assigned in the same orthogroup, with the birch gene in the same subclade as the *TBL41* being partially annotated and aligning poorly with the other sequences. *BpTBL3/31* is the only one of the *BpTBL29-35* genes associated with xylan assigned to cluster 9 (Figure 2B), with the remaining genes not clustered. However, for all of those TBLs associated with xylan SCW, but the *BpTLB35*, the expression peaks in the SCW are pronounced and all but *BpTBL35* are in cluster 6 with WGCNA. *BpTBL35* expression has a higher peak in F2 and another peak in F5, which suggests that the gene might be involved in PCW acetylation. The phylogenetic trees of the biosynthesis genes are included in supplementary materials.

**Notes S5** **Xylan MS analysis**

We correlated the clusters and individual genes with the MS peaks (**Figure 6**) and examined the genes with absolute correlation above 0.7. The reported peaks represent the intensity ratio of the main peak to that of internal standard. Clusters 1 and 19 were correlated with the PCW peaks, P4Ac1and P5Ac1, whereas clusters 5-7 and 12,13 (Figure 2B) were positively highly correlated with the SCW peak P4Ac2. Among the genes with correlation above 0.9 with the SCW peak, there is the ortholog of the Arabidopsis *XND1* – involved in programmed cell death and xylem development*. XND1* inhibits the SWNs activation of the downstream TFs involved in the synthesis of the SCW. It was found that this happens while cell elongation still takes place, after which the inhibitory activity of the *XND1* is no longer observed. This is an indicator of the role of *XND1* ensuring the growth of the vessels before the onset of SCW thickening and deposition driven by the NAC TFs. By transactivation analysis, XND1 was found to inhibit the activity of both *VND6* and *NST1* genes, and it was suggested that *NST1* forms a negative feedback loop with the *XND1* to regulate the initiation of the cell wall formation (Zhong et al., 2021). In addition, among the top correlated genes, multiple involved in (xylem) cell differentiation and vascular development (orthologs of MYBS2, WRKY12, ACL5 and HB8), and the ortholog of *MAP70-5* regulating cell wall patterning in Arabidopsis wood cells. Two MYB transcription factors, homologs of MYB52 and MYB54 - involved in regulation of SCW thickening, also correlate with the peak. In addition, multiple genes are involved in xylem fiber and vessel development and cell wall kinases in the list of correlated genes (Table S26). Even though the xylan biosynthesis genes, including those for the modifications, have SCW specific pattern, the xylan that is deposited with structure most abundant in the xylem tissues, has the distribution that highly corelates to the genes controlling the activity of the SCW master regulators, and potentially in vascular development and cell wall thickening, as suggested.

The main peak in the young phloem related to PCW was correlated with genes enriched for a wide range of stress responses. Looking at the genes individually, the PCW acetylation homolog of TBL 27 was on the list (Table S26). More detailed analysis of these genes could potentially lead to new candidates involved in the biosynthesis of PCW xylan.

The PCA analysis of the acidic POS from all tissue-specific wood fractions revealed similar observations as phloems and cork cambium were clearly separated from xylems by component 1 (75% variance; **Figure S27b**). The young phloem exhibited the most structurally distinct profile since they were completely separated from xylem by component 1. The loading plot of component 1 showed that phloem and cork cambium was dominated by short POS fragments, MeGlcA-P_3-5_, which were either non-acetylated or carrying one and two acetyls (**Figure S27c**). Besides, young and matured xylem were spreading across component 2 (variance 11%), and the loading (**Figure S27c**) showed that the acidic POS of the primary xylem were richer in highly acetylated MeGlcA-P_5-7_, implicating acetyl modification is possibly taking place during xylem maturation (**Figure S27c**).

**Notes S6 The main peak in hydrolysate of young phloem lacked acetyl substitution in the MeGlcA-linked xylose residue**

The young phloem had exhibited distinct acidic POS profile in the OLIMP analysis, especially the main peak observed was MeGlcA-P_4_Ac, which differed from that of xylems. To elucidate the positions of the MeGlcA and acetyl substitutions, the corresponding peak was selected for tandem MS (MS^n^) analysis. Fragmentation of underivatized oligosaccharide in negative ion ESI-MS produces predominantly C-type ions that enable stepwise sequencing of glycosyl residues from the reducing end (Chong et al., 2015).

In the MS^2^ analysis of MeGlcA-P_4_Ac (*m/z* 777; **Figure S27**), the fragment ions, ^0,2^A_5_ and ^0,2^A_5_ – H_2_O resulting from loss of 60 Da and 78 Da, respectively; were diagnostic of a non-substituted (1→4) linked pentose residue at the reducing end. While in the MS^3^ analysis, the ^*0,2^A_4_-ion produced from loss of 102 Da from C_4_-ion indicated the second pentose residue from the reducing end was substituted by an acetyl residue. The detection of C_1α_-ion and ^0,2^X_2_-ion in the MS^4^ spectrum were diagnostic of a MeGlcA substitution at the non-reducing end of the xylotetraose. The result showed that, compared to xylem, the main peak in young phloem lacked an acetyl substitution on the MeGlcA-linked xylose residue.

**Notes S7 The disaccharide side branch pentose-MeGlcA was detected in young phloem xylans**

Besides exhibiting a different main peak, the young phloem produced also shorter fragments compared to xylems. The longest acidic POS from young phloem comprised of five pentoses that were either non- or mono-acetylated. Intriguingly, if they were formed by xylopentaouronic acid, the residing substituents wouldn’t have been substantially restrictive to the GH10 endoxylansase degradation, therefore shorter fragments should be produced instead. This was proven in the study of GH10 endoxylansase by Biely et al. (1997) that the enzyme was reportedly producing MeGlcA-Xyl_3_ as main product, with the MeGlcA being located on the non-reducing end of the xylotriose. Nevertheless, longer fragment, MeGlcA-Xyl_4_ can be produced as minor product with an additional xylose residue residing either on the non-reducing end or reducing end of MeGlcA-Xyl_3_ (Chong et al., 2015). Therefore, the structure of MeGlcA-P_5_ in young phloem can be possibly formed by xylotetraouronic acid that carries an arabinose residue or an Ara-MeGlcA disaccharide side branch that has been previously found in the xylans of Arabidopsis and non-commelinid monocots (Chong et al., 2015; Mortimer et al., 2015).

To investigate further the positional specific of the pentose substitution in the MeGlcA-P_5_, the corresponding peak in young phloem was selected for MS^n^ analysis. The MS^n^ analysis of MeGlcA-P_5_, however, did not produce satisfactory spectra due to low abundancy, thus MeGlcA-P_5_Ac was analyzed instead. In the MS^2^ analysis (**Figure S30**), the ^0,2^A_6_- and C_5_-ion resulting from loss of 60 Da and 132 Da, respectively; suggested a (1→4)-linked non-acetylated Xyl residue on the reducing end of the oligosaccharide. The C_5_-ion (*m/z* 777) was chosen for MS^3^ analysis and the detection of C_4_- and ^*^C_4_-ion due to loss of 132 Da and 174 Da, respectively; indicated two possible isomeric structures, of which the second Xyl residue from the reducing end can be either non- or mono-acetylated. The C_4_- (*m/z* 645) and ^*^C_4_- (*m/z* 603) ion were then separately selected for MS^4^ analysis, loss of 132 Da that yielded C_3_- (*m/z* 513) and *C_3_- (*m/z* 471) ion suggested the third Xyl residue from the reducing end of both isomeric compounds were non-acetylated. Furthermore, ^0,2^X_3_- (*m/z* 381) and C_2α_-ion (*m/z* 339) that concomitantly detected in the MS^4^ spectra of both isomeric structure indicated a possible pentose-MeGlcA disaccharide side branch located at the non-reducing end of xylotetraose.

### **Notes S8 PACE analysis revealed primary wall-like MeGlcA substitution pattern and Ara-MeGlcA side branch in young phloem xylans**

Though the MS^n^ analysis suggested the occurrence of pentose-MeGlcA dissacharide side branch in the xylans from young phloem, the analysis was not able to completely exclude a possible pentose substitution that directly links to the oligosaccharide backbone like those detected in the primary wall of leaf tissues or cell suspension (McNeil et al., 1984, Zablackis et al., 1995). For further analysis, the AIR samples from young phloem and xylem were deacetylated with alkaline solution, digested with *Bo*GH30 xylansase, and the hydrolysates were then separated on carbohydrate gel electrophoresis (PACE). The method previously has been used to discover two (Me)GlcA decoration domains in the secondary wall xylans of Arabidopsis (Bromley et al., 2013), as well as an Ara-GlcA dissacharide side branch in the xylans from primary wall rich tissues from Arabidopsis stem (Mortimer et al., 2015). The enzymatic action of GH30 xylansase strictly requires a (Me)GlcA residing on the -2 subsite, thus the hydrolysis of xylans generally produces a series of xylooligosaccharides harboring (Me)GlcA on the second Xyl residue from the reducing end (Vršanská et al., 2007). As expected, the hydrolysis of young xylem that rich in secondary wall produced a series of MeGlcA linked xylooligosaccharides that co-migrated with that produced from Arabidopsis stem tissue (**Figure S33**). In contrast, the hydrolysis of young phloem produced mainly two bands on PACE gel, the most intense band migrated alongside MeGlcA-Xyl_6_, while the other one that present between Xyl_5_ and Xyl_6_, was identical to that previously identified as PUmeX_6_, which consisted of Ara-MeGlcA dissacharide side branch linked to a Xyl_6_ (Mortimer et al., 2015).

### **Notes S9 Two-dimensional NMR analysis confirmed lower acetylation level and an α-Ara*p*(1→2)-α-MeGlc*p*A side branch in the xylans from young phloem**

To analyze in detail the acetylation pattern, as well as the nature of the pentose substitution in the pentose-MeGlcA dissacharide side branch, acetylated and deacetylated xylans were fractionated from AIR samples, hydrolyzed by *Tr*GH11 endoxylansase and subjected to 2D NMR analysis. The acetylation pattern in the *O*-acetylglucuronoxylans was examined with quantitative HSQC analysis and the assignment of cross peaks were according to (Teleman et al., 2002, Chong e al., 2014). The degree of acetylation (DA) detected in the young xylems was 32% (Table S24), which was slightly lower than previously reported 40% DA in the xylans from birch whole wood (Teleman et al., 2002). Compared to young xylem, the DA in young phloem was decreased to 28% and the discrepancy was mainly attributed to reduction in 2-*O*-acetyl-Xyl (X2) and 3-*O*-acetyl-Xyl (X3), as well as 2,3-*O*-acetyl-Xyl (X23) (Table S24). In addition, the relative content for the α-MeGlcpA(1→2)-β-Xyl*p* (XG2) lacking acetyl substitution was also found to be enriched in the young phloem. The results were in good agreement with OLIMP analysis confirming that xylans from young phloem was typically deficient in acetyl substitutions.

Apart from differences in acetylation pattern, a H1/C1 cross peak at 5.39/97.83ppm that possibly corresponding to 2-α-MeGlc*p*A (Pena et al., 2016) was detected in the qHSQC spectrum of young phloem (**Figure S31**). To further investigate the possible pentose substitution in the MeGlc*p*A side group in young phloem, the deacetylated xylans isolated with alkaline solution were hydrolyzed by GH11 endoxylansase and analyzed with HSQC, TOCSY, COSY and NOESY. The non-hydrolyzed polymers were precipitated in 80% ethanol prior to NMR analysis to circumvent interferences from overlapped signals. The assignment of cross peaks in the 2D NMR spectra were according to (Peña et al., 2007, Pena et al., 2016).

Indeed, there were H1/C1 cross peaks at 5.41/97.87ppm and 4.60/106.08 ppm corresponding to 2-α-MeGlc*p*A and α-Ara*p*, respectively; found in the HSQC spectrum of young phloem (**Figure S32**). In addition, two variations of H1/C1 signals of 2,4-β-Xyl*p* at 4.62/102.71ppm and 4.68/102.55ppm indicated some Xyl*p* were attached to α-MeGlc*p*A and α-Ara*p*-(1→2)-α-MeGlc*p*A, respectively. The inter-glycosidic linkages were examined by NOESY measurement. The H1/H2’ signal that clearly detected at 5.41/3.54ppm established (1→2) linkage between 2-α-MeGlc*p*A and 2,4-β-Xyl*p* (**Figure S34**). Though the region comprised of signals for α-Ara*p* were heavily overlapped, there was nonetheless a weak H1/H2’ signal corresponding α-Ara*p*-(1→2)-α-MeGlc*p*A detected at 4.59ppm/3.74ppm. Taken together, the results from tandem mass spectrometry, PACE and 2D-NMR analysis confirmed that the dissacharide side branch, α-Ara*p*-(1→2)-α-MeGlc*p*A, was indeed present in the xylans from young phloem. It was estimated that 25% of the MeGlc*p*A in xylans from young phloem were substituted with α-Ara*p* based on qHSQC analysis.

**References**

| Wang, L., Wang, W., Wang, Y. Q., Liu, Y. Y., Wang, J. X., Zhang, X. Q., ... & Chen, L. Q. 2013. Arabidopsis galacturonosyltransferase (GAUT) 13 and GAUT14 have redundant functions in pollen tube growth. *Molecular Plant* 6: 1131-1148. |
| --- |
|  |

Sundell D, Street NR, Kumar M, Mellerowicz EJ, Kucukoglu M, Johnsson C, Kumar V,

Mannapperuma C, Delhomme N, Nilsson O, Tuominen H. 2017. AspWood: high-spatial-

resolution transcriptome profiles reveal uncharacterized modularity of wood formation in

Populus tremula. *The Plant Cell* 29: 1585-1604.

Brown D, Wightman R, Zhang Z, Gomez LD, Atanassov I, Bukowski JP, Tryfona T, McQueen‐Mason SJ, Dupree P, Turner S. 2011. Arabidopsis genes IRREGULAR XYLEM (IRX15) and IRX15L encode DUF579‐containing proteins that are essential for normal xylan deposition in the secondary cell wall. *The Plant Journal*, 66: 401-413

Manabe Y, Nafisi M, Verhertbruggen Y, Orfila C, Gille S, Rautengarten C, Scheller HV. 2011. Loss-of-function mutation of REDUCED WALL ACETYLATION2 in Arabidopsis leads to reduced cell wall acetylation and increased resistance to Botrytis cinerea. Plant Physiology 155: 1068-1078.

Wang Y, Xu Y, Pei S, Lu M, Kong Y, Zhou G, Hu R. 2020. KNAT7 regulates xylan biosynthesis in Arabidopsis seed-coat mucilage. *Journal of Experimental Botany* 71: 4125-4139.

Qin W, Yin Q, Chen J, Zhao X, Yue F, He J, Yang L, Liu L, Zeng Q, Lu F, Mitsuda N. 2020.

The class II KNOX transcription factors KNAT3 and KNAT7 synergistically regulate

monolignol biosynthesis in Arabidopsis. *Journal of Experimental Botany* 71: 5469–5483.

Cosgrove, D. J., & Jarvis, M. C. 2012. Comparative structure and biomechanics of plant

primary and secondary cell walls. *Frontiers in plant science* 3: 204.

Li, Y., Jones, L., & McQueen-Mason, S. 2003. Expansins and cell growth. *Current opinion in*

*plant biology* 6: 603-610.

Rose JK, Saladié M, Catalá C. 2004. The plot thickens: new perspectives of primary cell wall

modification. *Current Opinion in Plant Biology* 7: 296-301.

Mortimer JC, Faria-Blanc N, Yu X, Tryfona T, Sorieul M, Ng YZ, Zhang Z, Stott K, Anders N, Dupree P. 2015. An unusual xylan in Arabidopsis primary cell walls is synthesized by GUX3, IRX9L, IRX10L and IRX14. The Plant Journal: For Cell and Molecular Biology 83: 413–426.

Wu AM, Hörnblad E, Voxeur A, Gerber L, Rihouey C, Lerouge P, Marchant A. 2010. Analysis

of the Arabidopsis IRX9/IRX9-L and IRX14/IRX14-L pairs of glycosyltransferase genes reveals

critical contributions to biosynthesis of the hemicellulose glucuronoxylan. Plant Physiology

153:542-554.

Yuan Y, Teng Q, Zhong R, Ye ZH. 2016. TBL3 and TBL31, two Arabidopsis DUF231 domain

proteins, are required for 3-O-monoacetylation of xylan. *Plant and cell physiology* 57: 35-45.

**Gille S, de Souza A, Xiong G, Benz M, Cheng K, Schultink A, Pauly M.** 2011. O-acetylation of Arabidopsis hemicellulose xyloglucan requires AXY4 or AXY4L, proteins with a TBL and DUF231 domain. The Plant Cell 23: 4041-4053.

**Urbanowicz BR, Peña MJ, Moniz HA, Moremen KW, York WS.** 2014. Two Arabidopsis

proteins synthesize acetylated xylan in vitro. The Plant Journal **80**: 197–206.

Xiong G, Cheng K, Pauly M. 2013. Xylan O-acetylation impacts xylem development and enzymatic recalcitrance as indicated by the Arabidopsis mutant tbl29. *Molecular Plant* 6: 1373-1375.

Zhong R, Kandasamy MK, Ye ZH. 2021. XND1 regulates secondary wall deposition in xylem

vessels through the inhibition of VND functions. *Plant and Cell Physiology* 62: 53-65.

**Chong SL, Virkki L, Maaheimo H, Juvonen M, Derba-Maceluch M, Koutaniemi S, Roach M,**

**Sundberg B, Tuomainen P, Mellerowicz EJ, Tenkanen M.** 2014. O-Acetylation of

glucuronoxylan in Arabidopsis thaliana wild type and its change in xylan biosynthesis mutants.

Glycobiology 24: 494–506.

Biely P, Vršanská M, Tenkanen M, Kluepfel D.1997. Endo-β-1, 4-xylanase families: differences

in catalytic properties. *Journal of biotechnology*, 57: 151–166.

Chong, S. L., Koutaniemi, S., Juvonen, M., Derba-Maceluch, M., Mellerowicz, E. J., &

Tenkanen, M. 2015. Glucuronic acid in Arabidopsis thaliana xylans carries a novel pentose

substituent. *International Journal of Biological Macromolecules* 79: 807-812.

McNeil M, Darvill AG, Fry SC, Albersheim P. 1984. Structure and function of the primary cell walls of plants. Annual Review of Biochemistry 53: 625-663.

Zablackis E, Huang J, Muller B, Darvill AG, Albersheim P. 1995. Characterization of the cell-

wall polysaccharides of Arabidopsis thaliana leaves. *Plant physiology* 107:1129-1138.

Bromley JR, Busse‐Wicher M, Tryfona T, Mortimer JC, Zhang Z, Brown DM, Dupree P. 2013.

GUX1 and GUX2 glucuronyltransferases decorate distinct domains of glucuronoxylan with

different substitution patterns. *The Plant Journal* 74: 423–434.

Vršanská M, Kolenová K, Puchart V, Biely P. 2007. Mode of action of glycoside hydrolase

family 5 glucuronoxylan xylanohydrolase from Erwinia chrysanthemi. *The FEBS journal* 274:

1666-1677.

**Teleman A, Tenkanen M, Jacobs A, Dahlman O.** 2002. Characterization of O-acetyl-(4-O-

methylglucurono) xylan isolated from birch and beech. Carbohydrate Research **337**: 373–377.

Peña MJ, Zhong R, Zhou GK, Richardson EA, O'Neill MA, Darvill AG, York WS, Ye ZH.

2007. Arabidopsis irregular xylem8 and irregular xylem9: implications for the complexity of

glucuronoxylan biosynthesis. *The Plant Cell* 19: 549-563.

Pena MJ, Kulkarni AR, Backe J, Boyd M, O’Neill MA, York WS. 2016. Structural diversity of

xylans in the cell walls of monocots. *Planta* 244: 589-606.
